# A Review about Functional Illiteracy: Definition, Cognitive, Linguistic, and Numerical Aspects

**DOI:** 10.3389/fpsyg.2016.01617

**Published:** 2016-11-10

**Authors:** Réka Vágvölgyi, Andra Coldea, Thomas Dresler, Josef Schrader, Hans-Christoph Nuerk

**Affiliations:** ^1^LEAD Graduate School & Research Network, University of TuebingenTuebingen, Germany; ^2^School of Psychology, University of GlasgowGlasgow, Scotland; ^3^Department of Psychiatry and Psychotherapy, University of TuebingenTuebingen, Germany; ^4^German Institute for Adult Education – Leibniz Centre for Lifelong LearningBonn, Germany; ^5^Department of Psychology, University of TuebingenTuebingen, Germany; ^6^Knowledge Media Research Center – Leibniz Institut für WissensmedienTuebingen, Germany

**Keywords:** functional illiteracy, literacy, illiteracy, dyslexia, adults

## Abstract

Formally, availability of education for children has increased around the world over the last decades. However, despite having a successful formal education career, adults can become functional illiterates. Functional illiteracy means that a person cannot use reading, writing, and calculation skills for his/her own and the community’s development. Functional illiteracy has considerable negative effects not only on personal development, but also in economic and social terms. Although functional illiteracy has been highly publicized in mass media in the recent years, there is limited scientific knowledge about the people termed functional illiterates; definition, assessment, and differential diagnoses with respect to related numerical and linguistic impairments are rarely studied and controversial. The first goal of our review is to give a comprehensive overview of the research on functional illiteracy by describing gaps in knowledge within the field and to outline and address the basic questions concerning who can be considered as functional illiterates: (1) Do they possess basic skills? (2) In which abilities do they have the largest deficits? (3) Are numerical and linguistic deficits related? (4) What is the fundamental reason for their difficulties? (5) Are there main differences between functional illiterates, illiterates, and dyslexics? We will see that despite partial evidence, there is still much research needed to answer these questions. Secondly, we emphasize the timeliness for a new and more precise definition that results in uniform sampling, better diagnosis, conclusion, and intervention. We propose the following working definition as the result of the review: functional illiteracy is the incapability to understand complex texts despite adequate schooling, age, language skills, elementary reading skills, and IQ. These inabilities must also not be fully explained by sensory, domain-general cognitive, neurological or mental disorders. In sum, we suggest that functional illiteracy must be more thoroughly understood and assessed from a theoretical, empirical, and diagnostic perspective.

## On the Importance of Literacy

### About Literacy

According to the recent literacy rate, 85% of the adult population in the world is literate, and therefore worldwide about 757 million people are illiterate (UNESCO, 2015). Large-scale assessments measuring literacy skills indicate that in developing countries, illiteracy is more prevalent, while in developed countries, functional illiteracy is more prevalent (Bhola, 1995, p. 18). According to the Organization for Economic Co-operation and Development (OECD), *literacy* is defined as follows:

“*Literacy is defined as the ability to understand, evaluate, use, and engage with written texts to participate in society, achieve one’s goals, and develop one’s knowledge and potential* (OECD, 2013, p. 59).” More detailed, find other institutions, e.g., UNESCO.

Literacy and basic knowledge cannot be clearly separated from each other. Even though the term “literacy” is a part of basic knowledge, it is a precondition as well as an outcome of basic knowledge. Literacy may refer to the ability to read and write, but also to application-oriented basic knowledge that develops during the whole lifetime, not only during school years (Nickel, 2007).

Formal literacy has increased over the last decades. For instance, while in sub-Saharan Africa there are still 29.8 million children who do not have access to education, this number represents a one-quarter decrease from 2000. In contrast, in Europe “only” 0.7 million of children had never attended school in 2011 (UNESCO, 2013). However, despite improvements in formal literacy, many people still have problems understanding formal texts. On the one hand, this is a problem because in today’s society, functioning literacy plays a significant role. It appears in every aspect of daily life, e.g., opening bank accounts, reading ingredients of food products, understanding medication or technical instructions, signing contracts, etc. (Cree et al., 2012). On the other hand, this leads to fewer educational and employment opportunities and hinders living a successful life.

Possessing literacy has many benefits for individuals, families, communities, and nations. The improvement in literacy levels has beneficial effects on individual (e.g., self-esteem), political (e.g., democratic values), cultural (e.g., cultural openness), social (e.g., children’s health), and economic (e.g., individual income) levels (UNESCO, 2006). On the other hand, functioning in a society without literacy becomes more difficult: those who cannot acquire basic literacy skills have fewer opportunities in every area of life (Cree et al., 2012).

### About (Functional) Illiteracy

So far, we have talked about literacy. However, many people do not achieve literacy because of inadequate schooling or even despite adequate schooling. On 1949, the United Nations Educational, Scientific, and Cultural Organization (UNESCO) set the generalized functionality of literacy. The acquisition of reading and writing was regarded as basic rights: people should be enabled to become functionally literate in their own culture (Bhola, 1995). A need for a standard and a workable definition materialized to differentiate between literates and non-literates (illiterates) and also to distinguish various levels in between. The result of the demand was realized at the General Conference of the UNESCO in 1978:

“A person is literate who can with understanding both read and write a short simple statement on his everyday life.

A person is illiterate who cannot with understanding both read and write a short simple statement on his everyday life.

A person is functionally literate who can engage in all those activities in which literacy is required for effective functioning of his group and community and also for enabling him to continue to use reading, writing, and calculation for his own and the community’s development.

A person is functionally illiterate who cannot engage in all those activities in which literacy is required for effective functioning of his group and community and also for enabling him to continue to use reading, writing, and calculation for his own and the community’s development (UNESCO, 1978, p.183).”

The difference between literate and illiterate people is explicit here: illiterates had never attended school and are unable to read or write even single words while literates can (Reis and Castro-Caldas, 1997).

In contrast with literacy and illiteracy, the difference between functional illiteracy, literacy and illiteracy is not obvious enough. Functionality, which is the essence of the difference between these terms, was never operationally defined. Recently, the number of functional illiterates in Europe was estimated to be about 80 million, their proportion is lowest in Sweden with 8% and highest in Portugal with 40% (e.g., in Eme, 2011; Grotlüschen and Riekmann, 2011a). However, the frequently referred original International Adult Literacy Survey (IALS) report does not imply functional illiteracy (OECD and Statistics Canada, 2000). Different definitions and different diagnostic assessment standards can lead to fundamentally different epidemiological estimations, so any estimations of functional illiteracy rates may be unreliable.

## Diagnostics of Functional Illiteracy: Different Approaches

As there is no explicit assessment for functional illiteracy, researchers had to find other techniques to assess the number of functional illiterates or to identify functional illiterates for experimental studies.

The UNESCO, the OECD and the IEA (International Association for the Evaluation of Educational Achievement) measure literacy and other key knowledge skills of children, young adults, and adults a large-scale, international assessment about strengths and weaknesses in different countries. Research such as the IALS and the Adult Literacy and Life Skills Survey (ALL) build on each other (Thorn, 2009; UNESCO, 2009). These kinds of international tests generally measure literacy and numeracy skills in various ways, including mapping the whole literacy spectrum and grouping the performance and the abilities into discrete levels. The international, supranational and national political actors are first interested in large-scale assessments, not in individual diagnostics. Against this background, it is understandable (but nevertheless at least unfortunate) that the diagnostic materials lack test criteria (reliability, construct validity, criterion validity), which are demanded in standard individual diagnostic tests.

The IALS, the ALL, and the PIAAC (Survey of Adult Skills) all contain *prose and document literacy tasks* that purport to understand and use information from different text formats. The *quantitative literacy* and *numeracy tasks* measure arithmetic abilities in all three assessments, but *problem solving tasks* are only included in the ALL and in the PIAAC study (**Table 1**). However, these studies usually analyze literacy in a theoretical way and give no practical diagnostic advice regarding the assessment of *functional* illiteracy. It can be only a conclusion from the result of the lowest achievement level.

**Table 1:** Summary of international assessments.

	Date	Number of countries	Tasks	Proficiency scales
IALS (Thorn, 2009)	1994–1998	23	1. Prose literacy 2. Document literacy 3. Quantitative literacy	Level 1–5
ALL (Statistics Canada and OECD, 2005)	2002–2006	12	1. Prose literacy 2. Document literacy 3. Numeracy 4. Problem solving	1. Level 1–5 2. Level 1–5 3. Level 1–5 4. Level 1–4
PIAAC (OECD, 2013)	2011–2012	24	1. Literacy (prose + document) 2. Numeracy 3. Problem solving in technology rich environments	1. Below level 1–5 2. Below level 1–5 3. Below level 1–3

A common way of diagnosing functional illiterates is based on the *years of schooling*. However, the standard seems to vary among cultures. In the USA, 12 years of schooling marks the limit of functional literacy (Bhola, 1995), while in Latin America, only 7 years of effective schooling is sufficient to exceed the level of functional illiteracy (Infante, 2000 In. Martinez and Fernandez, 2010). In the European Union, the compulsory education is between 9 and 13 years, so children can leave school between age 14 and 18 (European Commission, 2014/2015). Therefore, we cannot consider compulsory education as the only diagnostic attribute of functional illiteracy.

Another common diagnostic practice is using *grade-equivalent scores* and *reading-level match designs*. This concept is concrete, easy to understand, and it does not require a new specific test because the researchers use general standardized assessments. This method is mostly used when low literate adults are assessed and compared with primary school children (Greenberg et al., 1997; Thompkins and Binder, 2003; Greenberg, 2007; Rüsseler et al., 2011; Grosche, 2012; Eme et al., 2014). Comparing children who have already acquired basic reading, writing, and mathematical skills with low literate (functional illiterate) adults could answer a few questions. The developmental differences between children and adults can cause problems in interpreting the results of such studies.

The German Federal Ministry of Education and Research (Bundesministerium für Bildung und Forschung) organized a national strategy to reduce the number of people who do not acquire basic literacy skills. To explore the problem, Grotlüschen and Riekmann constructed a representative household survey, the Level One Study (leo.). They specified five alpha-levels a priori in the lea. (Literalitätsentwicklung von Arbeitskräften; Grotlüschen et al., 2011), however, the validity of these five alpha-levels (even their eventual borders) has not yet been – to the best of our knowledge – never systematically evaluated in a diagnostic manner. Nevertheless, these five levels were applied to the leo. The lea. was constructed to measure employees’ different competence domains, including literacy and aimed to support individual teaching and development instead of comparing a person to a social norm (Grotlüschen et al., 2011). The leo. aimed to assess people on the lower end of the literacy spectrum. The authors identified functional illiterates as those who perform in the first, second, or third level in the leo. According to their results, 14.5% of the working-age population (about 7.5 million people) in Germany is functionally illiterate (Grotlüschen and Riekmann, 2011a). It is important to note that 3.1 million adults (41%) of the estimated functional illiterate population were not native German speakers (Grotlüschen et al., 2014). This is a point which we view as critical, because despite general reading and writing skills, we are all functional illiterates in most foreign languages. In our view (outlined below), language production and comprehension do not need to be that of a native speaker, but should at least be mastered without major problems before a specific deficit in functional illiteracy can be diagnosed. Otherwise, what seems to be a fundamental reading problem is simply a problem of not mastering sufficiently a foreign language. Finally, and unfortunately, the test lacks multivariate analyses of construct validity and only descriptive statistics are available. Consequently, results and conclusions have to be interpreted with caution.

The authors suggest the individual differences resulting from various social roles make it impossible to create a general functional illiteracy test. They argue that different skills are required, for example for a highly qualified IT expert or a motor mechanic (Grotlüschen and Riekmann, 2011b).

For specific professions this is a valid argument, but it also raises the question of whether a general construct of literacy exists. To return to the example, in everyday life, IT experts and motor mechanics have to operate machines (e.g., laundry machine), have to read their bank statements, have to take medicine (and read package inserts), have to compare prices in the supermarket, etc. Therefore, we assume that some basic functional literacy skills should exist.

While the leo. is not considered a universal instrument for functional illiteracy by its authors, the Tests of Adult Basic Education (TABE) is a universal instrument to assess the mastery of basic skills and skills-growth measurement. The test includes practical, life-skills stimuli in an adult-relevant context (life-skills, work, and education) and contains tasks from the very low literacy level (e.g., recognizing letters, signs) to the advanced level (CTB/McGraw-Hill, 2008). The comprehensibility of the measured skills and the universality of the tasks suggest that it is possible to create an assessment to measure functional illiteracy, despite the fact that the main aim of the assessment is different.

It is important to note that functional illiterates (as low literate adults) would show floor effects in standard adult literacy (AL) and text comprehension tasks. This would make appropriate identification and within-group distinctions impossible. Therefore, it is worth considering the application of standardized tests for children to measure functional illiteracy. On the one hand, Egloff et al. (2011) argue for a *competence-based approach* to identify functional illiterates instead of a *norm-oriented view*. They suggest that it would be better to take different social expectations into account and handle the category of functional illiteracy as a less static phenomena. But on the other hand, they accept to use reading and spelling tasks (with child norms) with well-defined cut-off values to classify functional illiterates (Egloff et al., 2011).

To sum up, many methods have been used to identify functional illiterates, but none of these methods are yet standardized and systematically diagnostically evaluated in a representative sample of functional illiterates and adults. Therefore, they cannot be considered adequate for measuring and identifying functional illiterates on the basis of the current data.

## Who is Defined as Functional Illiterate?

Functional illiteracy is assumed to originate from cognitive or linguistic disorders and/or be associated with a sociocultural disadvantage (Eme, 2011; Boltzmann and Rüsseler, 2013). The diagnostic assessments and therefore the definition of the sample in different studies is not consistent and sometimes not even explicit.

For a rough categorization, we can divide sample definition of functional illiteracy in scientific publications into three groups:

(1) Some studies call their sample “functional illiterates,” but do not give any reason/explanation/diagnostic justification (Van Linden and Cremers, 2008; Kosmidis et al., 2011). From an educational-psychological perspective, it is not acceptable to categorize a subgroup without any empirical reason for doing so.
(2a) Some studies conduct experiments on adults taking part in basic courses [AL or adult basic education (ABE) classes] and call them functional illiterates (Thompkins and Binder, 2003). The similarity between functional illiterates and AL or ABE students is appropriate but has its shortcomings. In particular, it is not evident why people take these courses. Did they have sufficient schooling and nevertheless did not learn to read and write? Did they have insufficient schooling for whatever reason without the chance to become literate? Do they have profound reading/writing problems or are they taking these courses for other reasons (e.g., because the job center recommends doing them)? In short, the problem is that we have no assessment of how severe their functional illiteracy problem really is and whether we are encountering functional illiteracy or real illiteracy due to insufficient schooling.
(2b) It should be noted that there is also another group of studies concerning those who conduct experiments on AL or ABE students but do not call them functional illiterates (Greenberg et al., 1997; MacArthur et al., 2010). Despite that, theoretical backgrounds and reviews (e.g., Eme, 2011) frequently use these articles, which point out one main limitation of the field.
(3) Only a German and a French research group made explicit how they determine functional illiteracy in their studies. From the German side, Grosche (2012) used reading-level match design is his dissertation and labeled those ABE students as functional illiterates, who performed in two standardized reading tests in the level of first–fourth grade children (Grosche, 2012). While Rüsseler et al. (2013) used German diagnostic reading and spelling tests and involved only those adults to their intervention study who performed worse than average fourth grade level (Boltzmann and Rüsseler, 2013; Boltzmann et al., 2013; Rüsseler et al., 2013)^1^. The French group measured five components: phonological processing, orthographic processing, sentence comprehension, reading speed, and reading comprehension. Those ABE students who performed below the third grade level were then classified as functional illiterates (Eme et al., 2010). Three problems stick:
(i) The deficits of adult groups are defined as (severe) developmental delays. This cannot be taken for granted; for many adult deficits, and even for dyslexia, different patterns of deficits and developmental delays have been observed.
(ii) Even if one accepts that functional illiteracy is merely developmental delay, there is an inconsistency as regards the severity of the delay. While Rüsseler et al. (2013) suggest lower performance than (average) fourth grade level, Eme et al. (2010) suggest a more severe performance deficit even below third grade level.
(iii) The components for defining functional illiteracy differ between studies: while Rüsseler and colleagues use reading and spelling tests (Boltzmann and Rüsseler, 2013; Boltzmann et al., 2013; Rüsseler et al., 2013), Eme et al. (2010) use a much broader range of test components. It is still unknown which approach is more valid. In most definitions, functional illiteracy is mainly about impaired understanding of texts. We suggest that diagnostic tests should operationalize this definition and focus on impaired understanding of texts, until other test components prove important for diagnostic assessment of functional illiteracy.

In sum, there is inconsistency in definition and assessment of functional illiterates in the scientific literature. There are only a few studies that include well-established methods in the fundamental sampling question. As the literature lacks a clear definition and clear assessment criteria, we use the term “functional illiterate” to refer to all the participants from the three groups of scientific papers.

### Factors Contributing to Functional Illiteracy – The Scientific Aspect^2^

Unfortunately, few studies^2^ investigated differential diagnostic properties of functional illiteracy. Although there are related deficits that may or may not be part of functional illiteracy depending on the definition and the assessment tool. Here, we focus on three of these related deficits: language-related deficits, general cognitive deficits, and deficits related to numerical abilities (Supplementary Tables S1–S3).

#### Language-Related Deficits

The few articles that assess the basic skills of their specific sample separately have shown that functional illiterates have phonological processing deficits. Their profile is more similar to children with developmental dyslexia than to typical elementary school children. Adults performed much worse in phonological tasks than children matched for reading-level (Greenberg et al., 1997; Thompkins and Binder, 2003; Grosche, 2012; Eme et al., 2014).

Functional illiterates’ spelling skills are also weak (Greenberg et al., 1997; Thompkins and Binder, 2003; Eme et al., 2014): They rely more on orthographic processes (Greenberg et al., 1997), although they may also have orthographic processing difficulties (Greenberg et al., 1997; Thompkins and Binder, 2003; Eme, 2006). A comparison with reading-level matched children showed that their vocabulary size is also smaller (Greenberg et al., 1997; Eme et al., 2014) and they are slower in naming tasks (Grosche, 2012). Although functional illiterates seem to be a heterogeneous group, on the whole they performed poorer in phonology than in morphosyntax and semantics, with their low performance in oral language tasks being reflected in their written abilities (Eme et al., 2014).

This issue is further complicated by the fact that functional illiterates may not be a homogeneous sample. Eme et al. (2010) suggested that functional illiterates can be divided into five subtypes according to their oral narrative abilities (Eme et al., 2010). However, when the same research group examined the relationship between reading, spelling, and oral language abilities in a later study, the cluster analysis showed four profiles (Eme et al., 2014). So, the subtyping problem is not resolved yet.

Other papers (Eme, 2006; Grotlüschen and Riekmann, 2011a; Rüsseler et al., 2011; Eme et al., 2014) mention that functional illiterates have problems in text understanding but only one study examined whether more fundamental factors cause this difficulty. The paper that compared matched normal readers with functional illiterates and children with reading and writing disabilities found that the perceptual skills of functional illiterates are weak but have no impact on reading abilities (Rüsseler et al., 2011).

In sum, functional illiterates seem to have linguistic deficits in several domains, including phonological, orthographic and lexical processing, oral and reading comprehension, and verbal fluency. However, these deficits may not be homogeneous. It is important to note that correlated or co-morbid deficits are not necessarily functionally causal. What is more, they do not necessarily add unique variance to the diagnostic assessment. Finally, we do not know whether the linguistic inabilities described above are their main difficulties or whether these are due to or influenced by other more general cognitive factors (Supplementary Table S1).

#### Cognitive Deficits

Cognitive deficits of functional illiterates have also been reported. Van Linden and Cremers (2008) showed that functional illiterates performed significantly worse than literates not only in language processing, but also in all cognitive tasks such as in copying and recalling the Rey Complex Figure, visual organizational, and visual memory, mental spatial orientation as well sustained or split attention tasks (Van Linden and Cremers, 2008).

Functional illiterates seem to have working memory difficulties: they performed worse than reading-level matched children (Eme, 2006; Grosche, 2012) and than normal adult readers (Grosche, 2012) in the verbal tasks. Comparing functional illiterates with children matched for reading-level, adults performed better on a backward, while they did not differ in a forward digit span task (Thompkins and Binder, 2003). However, the studies only used digit or letter span tasks (Thompkins and Binder, 2003; Eme, 2006; Grosche, 2012).

As regards perceptual skills, functional illiterates perform similar to children with reading and writing disabilities and differ from regular adult readers. This supports a developmental delay view on functional illiteracy (Rüsseler et al., 2011). The authors suggest that perceptual training could develop functional illiterates, as it improved the reading and spelling performance of children with reading and writing disabilities (Rüsseler et al., 2011).

In sum, it is clear that functional illiterates deviate from adults; their performance seems to be more similar to children. However, basic control variables (e.g., intelligence) are often missing, when the cognitive abilities of functional illiterates are assessed. Moreover, again participant selection could drive the results and the subsequent interpretations of deficits. Nevertheless, the available data point to the view that functional illiterates seem to show various cognitive deficits. However, the question about whether these deficits are (partially) causal for the functional illiteracy or just co-morbid impairments remains unanswered so far (Supplementary Table S2).

#### Deficits Related to Numerical Abilities and Dyscalculia

Although numerical abilities are measured as one of the basic skills and are considered as part of functional illiteracy (e.g., in IALS as quantitative literacy, Thorn, 2009; in ALL and in PIAAC as numeracy, Statistics Canada and OECD, 2005; OECD, 2013), research on numerical deficits in functional illiteracy has largely been neglected (Supplementary Table S3). Therefore, further experimental studies are needed to answer the question whether functional illiterates have numerical difficulties or not.

### (Functional) Illiteracy Programs – The Practical Aspect

In order to eradicate illiteracy, governments, NGOs (non-governmental organization) and supranational agencies such as UNESCO fund numerous programs worldwide (Abadzi, 2003), but the programs are assessed with great skepticism in the literature (Shi and Tsang, 2008). It is important to note that the ABE programs are rarely targeted explicitly at functional illiterates, as they generally aim to increase the participants’ literacy skills^3^.

In Western societies, adult literacy programs are often offered to vulnerable or hard-to-reach learners. Some programs rely extensively on the use of technology and distance learning platforms (e.g., AlphaRoute in Canada), others are tailored to each participant’s needs, both in workshops and individual help (e.g., Fight Against Illiteracy in France). According to their main interest, we can differentiate from general literacy courses the work- (e.g., El Trabajo En Red Como Proyecto Educativo in Spain) and family-oriented (e.g., Family Literacy Project in Germany) programs (Aker et al., 2010). Former supports the (re)integration to labor market (Bhola, 1995), while latter’s key-strategy called the “Teach the parents – reach the children” approach in which parents and their children are working both separately and together. It aims at a long-term effect in the education of next generation (Nickel, 2007). Furthermore, supplementing literacy and numeracy classes with technology, even mobile phones, is restricted by its reduced availability (Aker et al., 2010).

Adult basic education classes are still struggling to overcome high drop-out rates, failure to pass literacy tests, and a fast deterioration of literacy skills. High drop-out rates are associated with younger age, worse blending, slower naming, and comprehension skills, as well as increased avoidance of reading difficult materials. Furthermore, current/past enrollment in ABE classes increased the probability of midpoint completion (Greenberg et al., 2012). Therefore, the programs should pay more attention to the participants that fall within these categories. In Germany, Rüsseler et al. (2012) created and investigated the effects of a special training program called Alpha Plus. While the regular literacy courses offer reading and writing classes once a week, the intensive Alpha Plus training does not only improve reading and writing skills. But it builds also on the progress of other basic, daily and work-related abilities (e.g., perceptual and social skills). The program is clearly more effective than the regular classes offered to functional illiterates by the adult education schools in Germany. The efficiency of Alpha Plus was confirmed by behavioral, ERP, and fMRI studies (Rüsseler et al., 2012; Boltzmann and Rüsseler, 2013; Boltzmann et al., 2013; Rüsseler et al., 2013). The success of the program is evident but the authors stress the large variability between the participants. The achievement would be larger if it could better handle individual differences (e.g., with more groups with smaller sizes; Rüsseler et al., 2013) and follow a more personalized adaptive learning approach.

To sum up, solving the problem of illiteracy and functional illiteracy is relevant to governments and various organizations and their efficiency show up in statistics (UNESCO, 2015). But the development of programs based on scientific research (e.g., Alpha Plus: Rüsseler et al., 2012) could improve the efficacy of the programs and the persistence of the students.

## Dissociating Functional Illiteracy From Illiteracy and Dyslexia

For establishing a solid picture about the construct of functional illiteracy, it is necessary to distinguish it from related constructs such as illiteracy and developmental dyslexia, and to define non-overlapping characteristics. Without such dissociation, functional illiteracy is just a new name for a deficit that is already part of other constructs.

### Functional Illiteracy and Illiteracy: What Does Functionality Mean?

Illiteracy is a well-defined phenomenon and the diagnostic criteria for this group are clear-cut. It has been investigated since the 1970s and researchers have investigated many characteristics of illiteracy (Huettig and Mishra, 2014). According to the original notion, the difference between functional illiterates and illiterates is that illiterates are unable to read, write, and understand short sentences. In contrast functional illiterates are unable to use their acquired literacy skills in daily life (UNESCO, 1978), e.g., to read and understand a medicine label or a bank statement, fill out a job application, compare the cost of two items and choose the item that offers the best value (Cree et al., 2012).

When we outline these studies, we focus on the same three related groups of deficits we distinguished for functional illiterates (Supplementary Tables S1–S3).

#### Language-Related Deficits in Illiterates

As the illiterates have never attended school and did not acquire basic language skills, they differ in most language-related abilities. It is known that phonemic awareness is not attained spontaneously, since associations of phonemes with graphemes emerge with reading acquisition (Morais et al., 1979). Indeed, performances on phoneme addition, discrimination, deletion, and pseudoword repetition tasks (e.g., Greenberg et al., 1997; Thompkins and Binder, 2003) clearly demonstrated that illiterates have phonological processing deficits (Morais et al., 1979; Rosselli et al., 1990; Reis and Castro-Caldas, 1997; Castro-Caldas et al., 1998).

Decreased performance was shown also in orthographic (Petersson et al., 2000) and in lexical processing (Kosmidis et al., 2006) when low literate and literate adults were compared.

In addition, researchers observed impairments in naming ability (Rosselli et al., 1990; Ostrosky-Solis et al., 1999; Reis et al., 2006), in oral comprehension (Rosselli et al., 1990; Ostrosky-Solis et al., 1999) and in verbal fluency skills (Rosselli et al., 1990; Reis and Castro-Caldas, 1997; Ostrosky-Solis et al., 1999; Kosmidis et al., 2004) as well. Yet, it is important to mention that when using ecologically more valid categories in the verbal fluency task (e.g., supermarket), the difference can disappear (Reis et al., 2003).

In sum, illiterates can be characterized by impairments in the whole spectrum of language-related skills (Supplementary Table S1), which are less variable than those of functional illiterates.

#### Cognitive Deficits in Illiterates

As lack of reading and writing acquisition affects language skills, could it be assumed that basic cognitive functions also depend on it? The need for assessing the cognitive abilities of illiterates materialized many years ago.

Illiterates performed significantly worse than the three other assessed educated groups (1–4; 5–9; 10–24 years of education) in abilities as orientation, verbal fluency, attention, perception, and motor functions (Ostrosky-Solis et al., 1999; Dansilio and Charamelo, 2005; Landgraf et al., 2011). The latter was confirmed in visuo-motor integration tasks as well: while literates used a systematic visual scanning strategy, illiterates were less systematic and slower in a computerized visual-motor task (Bramão et al., 2007).

Oral cultures have better long-term memory abilities, as they can preserve their traditional songs by rote learning (Huettig and Mishra, 2014). Conversely, illiterates did not succeed in standardized working memory tasks (Ardila et al., 1989; Reis et al., 2003; Kosmidis et al., 2011; Silva et al., 2012). In addition, Kosmidis et al. (2011) revealed that literacy *per se* and not formal schooling affected working memory skills.

In sum, illiterates perform worse in various cognitive skills than literates. The deficits seem more universal than in studies with functional illiterates. Lack of education and basic skill acquisition have been brought forward as the reason for the weakness of cognitive skills in illiterates (Ardila et al., 1989; Rosselli et al., 1990) (Supplementary Table S2).

#### Deficits Related to Numerical Abilities in Illiterates

Although illiterates never attended school and never acquired number reading and writing, the majority of the tests that examine mental calculation or basic arithmetical abilities were administered to illiterates in written form. It is not surprising that these studies solidly verified that illiterates have poor mental calculation or basic arithmetical abilities (Ostrosky-Solis et al., 1999; Reis et al., 2003; Landgraf et al., 2011; Silva et al., 2012). Only one experiment gave calculations orally where the illiterates achieved low score as well (Rosselli et al., 1990). However, it is also possible that the deficits extend to basic number sense. Halberda and Feigenson (2008) have shown that early processing of non-symbolic information long before formal schooling influences arithmetic performance at a later age (Halberda and Feigenson, 2008). Whether the so-called approximate number system (ANS) – measured by non-symbolic magnitude comparison – really contributes to symbolic and arithmetic performance when other symbolic factors are controlled is a matter of intense discussion (De Smedt et al., 2013; Lyons et al., 2015). The answer to this question is not easy as performance in ANS tasks and their correlations with arithmetic seem to depend on the particular method involved (Dietrich et al., 2015). Nevertheless, it would be helpful to assess more basic numerical abilities like the ANS or spatial-numerical capabilities (Siegler and Opfer, 2003; Moeller et al., 2009) or indices of multi-digit integration (Moeller et al., 2011; Nuerk et al., 2015 for a review) to identify basic numerical deficits in functional illiterates that might lead to deficits in later more complex arithmetic tasks.

In sum, illiterates performed less accurately not only in language-related tasks, but also in cognitive and mathematical tasks. But it remains unclear whether the lack of reading acquisition, the absence of formal education, or even basic perceptual and cognitive deficits underlying more than one skill drive their functional illiteracy (Supplementary Table S3).

### Functional Illiteracy and Dyslexia: Different Constructs for the Same Sample?

Is it possible that functional illiterates are dyslexics with a new name?

We have outlined above that various language deficits are part of functional illiteracy. Some authors even claim that functional illiterates can somehow count as untreated developmental dyslexics (Greenberg et al., 1997; Grosche, 2012, but see diagnostic problematic outlined above). Therefore, it is unclear whether the terms “functional illiterate” and “dyslexic” reflect different terminology used to refer to the same group of people due to preference and history of the field, rather than due to actual differences between the two groups. It is surprising that we have not found any experimental research that has investigated this thesis. Therefore, we will outline developmental dyslexia in more detail, again with the same three subsections, language-related deficits, general cognitive, and numerical deficits (Supplementary Tables S1–S3).

#### Language-Related Deficits in Dyslexia

Developmental dyslexia is associated with abnormalities in a variety of brain regions, and has a strong genetic basis (Lyon et al., 2003; Fletcher, 2009; Habib and Giraud, 2013). However, it is not clear whether the neurobiological changes are a cause or consequence of reading difficulties.

Dyslexic children have problems in at least three domains: decoding single words, reading fluency, and comprehension (Fletcher, 2009). Leading theories suggest that the main problem in dyslexia is the phonological processing deficit. It can appear even at a single word level, independently of intelligence and is adequate for a dyslexia diagnosis (Ramus et al., 2003). Such a deficit in phonological awareness was confirmed in children by many studies (Joanisse et al., 2000; Casalis et al., 2004; White et al., 2006; Everatt et al., 2008; Landerl et al., 2009; Varvara et al., 2014; Zoubrinetzky et al., 2014). The most common tasks were phonological fluency (Landerl et al., 2009; Varvara et al., 2014) and manipulation with phonemes as phoneme deletion (Joanisse et al., 2000; Landerl et al., 2009; Chung et al., 2010; Zoubrinetzky et al., 2014) and spoonerism tasks (White et al., 2006; Varvara et al., 2014).

The results suggest that the phonological symptoms associated with dyslexia persist into adulthood (Hatcher et al., 2002; Ramus et al., 2003; Beidas et al., 2013; Bogdanowicz et al., 2014; Law et al., 2015). A study that compared adults with and without learning difficulties demonstrated that even high-achieving dyslexic adults are slower in phonological, semantic, and syntactic judgment tasks (Rüsseler et al., 2007).

In spelling, the tendency remains similar: both dyslexic children (White et al., 2006; Everatt et al., 2008; Chung et al., 2010) and adults (Hatcher et al., 2002; Beidas et al., 2013; Law et al., 2015) showed difficulties in their performance. In contrast, dyslexic adults performed well in the semantic fluency task (Hatcher et al., 2002) and vocabulary tasks (e.g., Cavalli et al., 2016) but the success of children were mixed (Joanisse et al., 2000; White et al., 2006; Everatt et al., 2008; Landerl et al., 2009; Varvara et al., 2014).

Nevertheless, their reading and naming speed were also significantly slower than in children and adults without learning difficulties (De Luca et al., 2002; Hatcher et al., 2002; Ramus et al., 2003; White et al., 2006; Everatt et al., 2008; Willburger et al., 2008; Boets and De Smedt, 2010; De Smedt and Boets, 2011; Beidas et al., 2013; Bogdanowicz et al., 2014; Suarez-Coalla et al., 2014; Law et al., 2015).

Basic language-related skills are necessary for accurate text comprehension (Martens and de Jong, 2006). Therefore, it is not surprising that dyslexic children and adults systematically perform below-average on reading comprehension tasks (Casalis et al., 2004; Fletcher, 2009; Rimrodt et al., 2009; Wiseheart et al., 2009; Rello et al., 2013). If texts are optimized according to word-frequency and word-length, thus using more common and shorter words, dyslexic adolescents and adults understand better and read faster written materials (Rello et al., 2013).

Dyslexia is not only categorized by phonological deficit, reading fluency, and text comprehension; it is also considered to be a heterogeneous learning disorder (Zoubrinetzky et al., 2014). Co-morbid language deficits and other cognitive difficulties are common. A regression study that aimed to examine the contribution of linguistic and cognitive factors to oral reading fluency in dyslexic adolescents found that word decoding, working memory, and vocabulary are the key predictors. The factors together explain 56% of the variance in connected-text oral reading fluency (Rose and Rougani, 2012). Despite the regression analysis, one must keep in mind that these are still correlations. Whether co-morbid cognitive difficulties causally influence reading fluency or whether linguistic deficits cause associated cognitive problems over the course of learning and development is not entirely clear yet (Beidas et al., 2013) (Supplementary Table S1).

In sum, we can conclude that dyslexic children have problems in phonological tasks, reading fluency, reading comprehension and associated linguistic and cognitive factors. Most such deficits observed in dyslexic children are preserved in adulthood. However, dyslexic adults may be able to compensate some of their deficits (e.g., in reading comprehension) and function better in language-related tasks than functional illiterates. Whether this summary of the literature holds, must be examined, with direct investigation of dyslexics and functional illiterates.

#### Cognitive Deficits in Dyslexia

In the last decades, auditory, visual processing, or attention deficits were suggested as being potential sources of dyslexia. Valdois et al. (2004) argue that phonological and attention deficits in dyslexic patients can present independently from each other (Valdois et al., 2004). Accordingly, dyslexics struggle with attentional and perceptual difficulties (Ramus et al., 2003; Ziegler et al., 2010; Leong et al., 2011; Beidas et al., 2013; Bogdanowicz et al., 2014; Varvara et al., 2014; Zoubrinetzky et al., 2014).

As regards cognitive abilities, most articles are examining working memory. It was shown that dyslexic children have poor working memory (Beneventi et al., 2010; Varvara et al., 2014), which remains weak during adulthood (Ramus et al., 2003; Abd Ghani and Gathercole, 2013; Beidas et al., 2013; Bogdanowicz et al., 2014). This deficit seems stable, considering that weak performance appears both in verbal (e.g., digit span, e.g., Everatt et al., 2008), in spatial (e.g., Corsi blocks, Landerl et al., 2009), and in visual (e.g., n-back, Beneventi et al., 2010) working memory tests. Exploring the four regions of executive functions (inhibition, planning, sequencing, and organizing abilities), researchers found that compensated dyslexic university students did not differ from the non-dyslexic control group in any of the functions (Brosnan et al., 2002). A more recent study showed that in a set shifting task, dyslexic adults were slower than age and IQ matched controls. In contrast, in an inhibition task the reaction time did not differ, although the accuracy depended on the task (Smith-Spark et al., 2016).

Experiments showed that dyslexic children have no problems in tasks requiring fine manual skills (White et al., 2006; Everatt et al., 2008) but they have difficulties in balancing tasks (White et al., 2006; Brookes et al., 2010). Conversely, adults did not show any problems in balance and motor coordination tasks (Ramus et al., 2003).

In sum, diverse types of cognitive difficulties are inseparable from the symptoms of dyslexia both in childhood and adulthood. Over time, dyslexics can improve some of their skills but most of their problems are remained. Nevertheless, their deficits seem less universal than in functional illiteracy (Supplementary Table S2).

#### Deficits Related to Numerical Abilities and Dyscalculia in Dyslexia

Research examining mathematical abilities has shown that dyslexic children and adults generally solved basic arithmetical problems slower and less accurately than children and adults without dyslexia (Hatcher et al., 2002; Simmons and Singleton, 2006; Boets and De Smedt, 2010; De Smedt and Boets, 2011).

A study examining children with reading disability and/or math disability found that all three groups showed difficulties in the examined neuropsychological measures. However, the impairments of reading and math disability group were the largest (Willcutt et al., 2013). Studies confirmed that reading and mathematical learning disabilities have independent domain-specific deficits: in the case of dyslexia in phonological processing and numerosity in the case of dyscalculia. Nevertheless, there are some common domain-general “bridge symptoms” as rapid naming (Wilson et al., 2015), working memory, processing speed, and verbal comprehension (Willcutt et al., 2013). In contrast, another experiment described that the cognitive deficits of children with dyslexia and dyscalculia were only additive (Landerl et al., 2009) (Supplementary Table S3).

The Triple-Code Model (Dehaene and Cohen, 1995) supposes three distinguished mental representations of numbers within different brain areas. According to the model, we can distinguish visual representation (established in the left and right inferior ventral occipito-temporal areas), magnitude representations (established in the left and right inferior parietal areas), and verbal representation (established in the left-hemispheric perisylvian language areas). Thus, the numerical and linguistic representations work separately. Therefore, those who have poor numerical or poor reading skills might be differentiated clearly according to their anatomical and functional brain processes (Dehaene and Cohen, 1995, 1998).

In sum, we can state that reading disabilities do not go obviously hand in hand with mathematical weaknesses, therefore, not just dyslexics and dyscalculics but also functional illiterates and functional innumerates may represent separate groups (Supplementary Tables S1–S3).

## Summary, New Definition, and Further Challenges

From the outline of the review, it is clear that the field of functional illiteracy has been under-represented in research despite its worldwide effects on social and economic levels (UNESCO, 2006) and although millions of dollars are invested in remediation programs of (functional) literacy.

In this review, we clarified our knowledge about functional illiterates, especially how different approaches try to diagnose them, and in what areas they differ from illiterates and dyslexics.

We summarized the challenges of empirical research that hinder the researchers of the field as the lack of an adequate assessment and resources for programs and researches.

A comprehensive, exploratory examination is needed to guarantee the success of the literacy programs. This examination should assess in detail the basic foundations and the variables that play a crucial role in functional illiteracy, emphasizing not only the language, but the mathematical-related and cognitive skills which are essential in everyday life.

The first step in that direction is to establish a new, up-to-date definition that is adequate for experimental research:

Functional illiteracy is the incapability to understand complex texts^4^ despite adequate schooling, age, language skills, elementary reading skills, and IQ. These inabilities must also not be fully explained by sensory, domain-general cognitive, neurological or psychiatric deficits.

Here we suppose the main criteria and justification that a working definition should contain:

Inclusion criteria:

– very poor performance in a functional illiteracy assessment: despite the fact that there is no consensus about an operationalized definition of functional illiteracy, many self-claimed assessments tried to measure it, but there is no standardized and validated tool for this aim^5^,
– age: older than 16 years old. We suppose that children cannot be categorized as functional illiterates,
– schooling: minimum 6–8 finished years, in agreement with the duration of compulsory education for single countries (in Germany it means 9 years),
– proper (German) language use: fluent, native-like oral language skills without major difficulties (natives, bilinguals). We should take with great care people with migration background because we cannot be sure whether a person shows weakness because he/she is a functional illiterate or because he/she has difficulties in second language acquisition. Nevertheless, being a native speaker is in our view not a necessary criterion if the second language is sufficiently well mastered in oral language,
– IQ: level of 70 or above.

Exclusion criteria:

– neurological or mental disorder,
– uncorrected speech, hearing, or vision problem.

Exclusion criteria for pure functional illiteracy:^6^

– dyslexia,
– dyscalculia,
– hyperactivity.

Further characteristics that describe functional illiterates:

– impaired oral language comprehension,
– impaired writing skills,
– impaired arithmetic skills,
– difficulties in functioning in society: problems with active, independent functioning in daily life.

Due to lack of empirical studies the underlying cause of functional illiteracy is still unclear. Rüsseler et al. (2011) suggested a combined model, where the unfavorable familiar background and school experiences could be identified as risk factors and together with biological and cognitive determinants could cause functional illiteracy (Rüsseler et al., 2011).

As regards our five research questions in the beginning, they can be answered as follows. We propose four different social and cognitive aspects that can lead to functional illiteracy in itself or together:

(1) Cognitive aspect: weak cognitive skills cause the inability to acquire proper basic literacy skills;
(2) Educational aspect: primary and secondary school teachers have no opportunity to take care the individual level of each student, therefore the children with feeble abilities or low motivation fall behind in long-term;
(3) Social aspect: the lack of an encouraging and motivating model in a child’s family for acquiring new skills, having new experiences, can lead to an unmotivated learning style in school;
(4) Competency loss aspect: loss of competencies in adulthood caused by a decrease of cognitive demands (Q4).

The focus on cognitive and social aspects does not preclude that some of them (e.g., the cognitive aspects) are neurobiologically routed.

The review shows that despite formal education, functional illiterates do not possess basic skills (Q1). This general deficit can be theoretically distinguished from the deficits associated with illiteracy and dyslexia; illiterates lack formal education, while functional illiterates have had some schooling and therefore may have advantages from this education. Additionally, dyslexia has genetic underpinnings while social factors seems to have stronger impact on the development of functional illiteracy (Q5), therefore their diagnostic and remediation processes may differ as well.

From the summary we cannot conclude in which abilities functional illiterates have the largest deficit, because we did not find any research that aimed at measuring their mathematical abilities (Q2). We suppose that functional illiterates have both numerical and linguistic deficits. According to the Triple-Code Model, the underlying representations work separately (Dehaene and Cohen, 1995, 1998) but we do not know any research that has tried to confirm this in a functional illiterate sample (Q3).

Summarizing our presumptions about functional illiteracy in details, we define as functional illiterates those adults who attended the compulsory years in education but could not acquire basic reading, writing, and calculation skills. Their impairments negatively affect their effective functioning in everyday life. In particular, functional illiterates have poor language skills (writing, reading, oral communication) (e.g., difficulty understanding a medicine label) as well as poor arithmetic abilities (e.g., inability to compare the price of two products) that generally influence everyday life situations (e.g., get the information from a timetable). People belonging to this group have average or below-average IQ levels and their difficulties cannot result from any other kind of neurological or psychiatric disorder, organic problem, non-verbal learning problem, general learning difficulty or hyperactivity. Of course, these criteria do not exclude co-morbidities with such other impairments.

## Conclusion

We would stress the need for methodologically more substantiated research, comparing basic linguistic, numerical and cognitive functions in normal readers, functional illiterates, dyslexic adults, and reading-level matched dyslexic children.

## Author Contributions

RV, TD, JS, and H-CN made the review design, RV and AC did the literature search, and RV, AC, TD, JS, and H-CN wrote the paper.

## Conflict of Interest Statement

The authors declare that the research was conducted in the absence of any commercial or financial relationships that could be construed as a potential conflict of interest.

## Footnotes

1 The authors explicitly wrote this criteria only in Rüsseler et al. (2013, p. 242) but as they speak about the evaluation of the same training program in Boltzmann and Rüsseler (2013) and in Boltzmann et al. (2013), we suppose that they used the same inclusion criteria.

2 Relevant studies for this review were identified by (1) carrying out a keyword search in EBSCOhost, PsycInfo, and Google Scholar. It was conducted for keywords functional illiteracy, illiteracy, literacy, adult dyslexia, child dyslexia, and several variations of these keywords and the basic abilities that we mention in Supplementary Tables S1–S3. (2) And we were conducting a manual search for references cited in relevant papers.

3 http://www.unesco.org/uil/litbase/?menu=7

4 Beyond this scope of review but we define complex text as comprising at least of two sentences with some conjunctions or subjunctions and propositional relations between these sentences. Questions concerning such tests should be impossible to answer on the basis of understanding one single sentence alone. For a more precise definition we suppose computer linguistically quantifiable measures about the readability and complexity of a text (e.g., after the methods of Vajjala and Meurers, 2014).

5 According to our opinion, functional illiterates in general have fundamental problems in text comprehension. Therefore, we suggest using tasks based on text comprehension, enhanced with an interview about their educational background. We agree with Boltzmann and Rüsseler (2013) that children’s tasks are well suited for assessing the functional illiterate sample, e.g., because of their complexity that admit of the differentiation and their short length that is not frustrating. However, these tasks have not yet been normed to low literate adults.

6 We would like to stress that we do not want to exclude functional illiterates with dyslexia, but we would like to raise awareness that further research should pay more attention to the related linguistic and numerical impairments. It is likely that functional illiterates meet the criteria for dyslexia and because of the dissociation between dyslexia and functional illiteracy merits further investigations.
