# Supplementary material for: A Review about Functional Illiteracy: Definition, Cognitive, Linguistic, and Numerical Aspects
Source: Front Psychol. 2016 Nov 10;7:1617. doi: 10.3389/fpsyg.2016.01617 (PMC5102880; doi:10.3389/fpsyg.2016.01617)
Supplement: Supplementary file 1 [file Table_1.pdf]

Supplementary Material

A Review about Functional Illiteracy:

Definition, Cognitive, Linguistic and Numerical Aspects

Réka Vágvölgyi*, Andra Coldea, Thomas Dresler, Josef Schrader & Hans-Christoph Nuerk*

*** Correspondence:**

Réka Vágvölgyi, reka.vagvoelgyi@lead.uni-tuebingen.de

Hans-Christoph Nuerk, hc.nuerk@uni-tuebingen.de

1 Supplementary Tables

Supplementary Table S1 | Summary of language related deficits of illiterates, functional illiterates, dyslexic adults and children

Group	Performance	Study	Sample characteristics			Task
			Experimental	Control	Match	
**1. Phonological processing**						
**Illiterates**	Deficit	Morais et al., 1979	30 illiterate adults (age: 38-60)	30 people who learned to read beyond the usual age (age: 26-60)		phoneme addition and phoneme deletion tasks
		Rosselli et al., 1990[1]	100 native Spanish illiterates with no formal education and with illiterate parents	100 Spanish native professionals with professional parents	age, gender, handedness	phonological discrimination, word repetition, phonological fluency (letters: f, s)
		Reis and Castro-Caldas, 1997	20 illiterate adults	10 literate adults	gender, cultural and social background	pseudo-word repetition, phonological fluency (letters: p, b)
		Castro-Caldas, 1998	6 illiterate adults (age: 60-70)	6 literate adults (age: 57-69)	gender, social and cultural background	pseudo-word repetition
		Ostrosky-Solis et al., 1999[2]	199 native Spanish illiterate adults (age: 16-85)	199 / 201 / 201 native Spanish participants with 1-4 / 5-9 / 10-24 years of schooling (age: 16-85)		phonological fluency (letter: f)
		Petersson et al., 2000	6 illiterate adults	6 literate adults with 4 years of schooling	gender	pseudo-word repetition
		Kosmidis et al., 2004	19 illiterate adults (age: 63-92)	20 literate/low literate adults with 1-9 years of schooling (age: 56-85) / 21 literate/high- educated women with minimum 9 years of schooling (age: 55-74)	age, gender / gender	phonological fluency

(Continued)

[1] The authors divided the participants into groups according to 3 variables. Here we used the most relevant grouping variable: educational level.
[2] The authors ranked the participants into 16 groups according to age and years of education. Here we focus on the years of education.

Supplementary Table S1 | Continued: Phonological processing

Group	Performance	Study	Sample characteristics			Task
			Experimental	Control	Match	
**Illiterates**	Deficit	Kosmidis et al., 2006	19 illiterate adults (age: 63-92)	20 literate/low literate adults with 1-9 years of schooling (age: 56-85) / 15 literate/high- educated women with minimum 10 years of schooling (age: 55-74)	age, gender / gender	pseudo-word repetition
**Functional Illiterates**	Deficit	Greenberg et al., 1997[3] (2)	72 native English adults from ABE classes (age: 21-45)	72 native English children from Grades 3 to 5	gender, race, reading level	nonword decoding: pseudo-word reading (Woodcock Reading Mastery Test-Revised), phoneme deletion, phoneme segmentation
		Thompkins and Binder, 2003 (2)	the 15 less and the 15 most skilled readers from 60 adults from ABE classes (age: 17-55)	30 control children chosen from a 99 pool (age: 5-8)	reading level	phoneme recognition, phoneme deletion, phonological spelling
		Eme, 2006 (3)[4]	50 native French functional illiterates (age: 17-55)	20 / 20 / 20 / 20 native French control children from preparatory class (CP) / “1st grade” (CE1) / “2nd grade” (CE2) / “4th grade” (CM2) (mean age: 6,7 / 7,6 / 8.8 / 10.7)		reading and writing pseudo-words[5], phoneme deletion[6], phoneme segmentation[7]
		Grosche, 2012 (3)	54 ABE students (age: 33-53)	66 native German control adults / 54 children from 1st to 4th grade (age: 32-53 / 7-9)	chronological age / reading level	vowel substitution, phoneme categorization, identification of vovels’ length[8] (Basiskompetenzen für Lese-Rechtschreibleistungen)

For explanation of (2), (3) see end of the table.

(Continued)

[3] Main effect of groups.
[4] Main effect of groups.
[5] Significant difference with CE1, CE2, CM2.
[6] Significant difference with CE2, CM2.
[7] Significant difference with CE1, CE2, CM2.
[8] Non-significant difference between functional illiterates and children.

Supplementary Table S1 | Continued: Phonological processing

Group	Performance	Study	Sample characteristics			Task
			Experimental	Control	Match	
**Functional Illiterates**	Deficit	Eme et al., 2014 (3)	52 native French ABE students (age: 17-55)	52 native French children from 1st to 3rd grade	reading level	pseudo-word reading, phonological recall (Wechsler Adult Intelligence Scale-III), phoneme deletion and inversion
	Normal	Eme, 2006 (3)[9]	50 native French functional illiterates (age: 17-55)	20 / 20 / 20 / 20 native French control children from preparatory class (CP) / "1st grade" (CE1) / "2nd grade" (CE2) / "4th grade" (CM2) (mean age: 6.7 / 7.6 / 8.8 / 10.7)		syllable deletion
		Grosche, 2012 (3)	54 ABE students (age: 33-53)	54 children from 1st to 4th grade (age: 7-9)	reading level	identification of vovels' length (Basiskompetenzen für Lese-Rechtschreibleistungen)
**Dyslexic Adults**	Deficit	Hatcher et al., 2002	23 dyslexic university students (age: 19-52)	50 university students (age: 18-41)	verbal and non-verbal abilities	nonsense passage reading, spoonerism, phonemic fluency (letters: m, d, s)
		Ramus et al., 2003	16 dyslexic university students (age: 19-22)	16 university students (age: 19-24)	age, gender, handedness, full-scale IQ	automatic digit naming, spoonerism, non-word repetition, non-word reading
		Rüsseler et al., 2007	11 native German high-achieving dyslexic adults (university students) (age: 19-30)	11 native German university students (age: 19-33)	age, gender, handedness	rhyme judgment
		De Smedt and Boets, 2011	25 dyslexic university students (age: 18-28)	25 normal reading controls (age: 18-29)	age, nonverbal IQ	phoneme deletion, spoonerism

For explanation of (3) see end of the table.

(Continued)

[9] Main effect of groups.

Supplementary Table S1 | Continued: Phonological processing

Group	Performance	Study	Sample characteristics			Task
			Experimental	Control	Match	
**Dyslexic Adults**	Deficit	Beidas et al., 2013	34 native Hebrew dyslexic university students (age: 21-28)	35 native Hebrew university students (age: 21-28)	chronological age, nonverbal IQ, verbal ability, handedness	One Minute Tests for Words and Non-Words, phonemic deletion from pseudo-words, segmentation
		Bogdanowicz et al., 2014	93 native Polish developmental dyslexic university students (age: 19-24)	87 native Polish university students (age: 20-24)		Unknown Language Test - part one
		Law et al., 2015	36 native English dyslexic university students (age: 17-26)	54 native English university students (age: 19-25)	age, gender, education, non-verbal IQ	spoonerism (onset-rhyme awareness, phoneme manipulation and deletion) (Phonological Assessment Battery)
		Wilson et al., 2015	18 / 22 / 26 native English dyslexic (a) / dyscalculic (b) / dyslexic & dyscalculic adults (c) (age: 30-32 / 27-31 / 28-32)	19 native English control adults (d) (age: 26-30)	age, gender, handedness, education, socio-economic status	Phoneme reversal (Comprehensive Test of Phonological Processing)[10]
**Dyslexic Children**	Deficit	Joanisse et al., 2000	61 dyslexic children (age: 7-9)	52 / 37 non-dyslexic children (age: 7-9 / 6-8)	chronological age / reading level	phoneme deletion and blending
		Casalis et al., 2004	33 native French dyslexic children (age: 8-12)	33 / 33 native French non-dyslexic children (mean age: 7.4 / 10.8)	chronological age / reading age	phoneme suppression
		White et al., 2006	23 dyslexic children (age: 8-12)	22 control children (age: 8-12)	age, gender, non-verbal IQ	rhyme, spoonerism, non-word reading (Phonological Assessment Battery)
		Everatt et al., 2008	20 native English dyslexic children (age: 11-12)	40 native English control children (age: 11-12)	age, gender	phonological segmentation (Dyslexia Screening Test)

(Continued)

[10] Significant difference between a and d.

Supplementary Table S1 | Continued: Phonological processing

Group	Performance	Study	Sample characteristics			Task
			Experimental	Control	Match	
**Dyslexic Children**	Deficit	Landerl et al., 2009	21 native German dyslexic children (a) (age: 7-10)	20 / 26 / 42 native German dyscalculic (b) / dyslexic & dyscalculic (c) / control children (d) (age: 7-10 / 7-11 / 7-10)	age	phoneme deletion[11]
		Willcutt et al, 2013	241 /183 / 188 children with reading disability (a) / math disability (b) / reading & math disability (c) (age: 8-13 / 9-13/ 8-13)	411 control children (d) (age: 8-13)	age, gender, ethnicity	phoneme deletion, pig latin[12]
		Zoubrinetzky et al., 2014	14 / 14 native French dyslexic children with visual attention span difficulties (a) / with phonological difficulties (b) (age: 8-12 / 8-11)	14 / 14 native French control children (c) / (d) (age: 8-11 / 6-7)	(c) chronological age / (d) reading age	phoneme deletion and segmentation, acronyms (Batterie d'évaluation du langage écrit et de ces troubles)[13]
		Varvara et al., 2014	60 children and adolescents with developmental dyslexia (age: 8-17)	65 children with typical reading abilities (age: 8-16)	chronological and mental age	spoonerism, phonological fluency (letters: f, a, s)
	Normal	Landerl et al., 2009	21 native German dyslexic children (a) (age: 7-10)	20 / 26 / 42 native German dyscalculic (b) / dyslexic & dyscalculic (c) / control children (d) (age: 7-10 / 7-11 / 7-10)	age	phonological fluency (letter: m)
		Chung et al., 2010	77 dyslexic students (age: 12-14)	27 / 27 normally achieving students (age: 12-14 / 9-13)	chronological age, IQ / reading level, IQ	phoneme onset deletion

(Continued)

[11] Significant difference between a and b, d.
[12] Significant difference between a, b and d.
[13] Significant difference between a and b, d; b and a, c, d; c and b, d; d and a, b, c.

Supplementary Table S1 | Continued: Orthographic processing

Group	Performance	Study	Sample characteristics			Task
			Experimental	Control	Match	
**2. Orthographic processing**						
**Illiterates**	No data					
**Functional Illiterates**	Deficit	Greenberg et al., 1997[14] (2)	72 native English adults from ABE classes (age: 21-45)	72 native English children from 3rd to 5th grade	gender, race, reading level	sight word reading, spelling inventory, rhyme word reading
		Thompkins and Binder, 2003 (2)	the 15 less and the 15 most skilled readers from 60 adults from ABE classes (age: 17-55)	30 control children chosen from a 99 pool (age: 5-8)	reading level	orthographic spelling
		Eme, 2006 (3)[15]	50 native French functional illiterates (age: 17-55)	20 / 20 / 20 / 20 native French control children from preparatory class (CP) / “1st grade” (CE1) / “2nd grade” (CE2) / “4th grade” (CM2) (mean age: 6.7 / 7.6 / 8.8 / 10.7)		dictation: in the level of phonology[16], lexical[17], morphology[18]
	Normal	Greenberg et al., 1997[19] (2)	72 native English adults from ABE classes (age: 21-45)	72 native English children from 3rd to 5th grade	gender, race, reading level	wordlikeness choice, letter position
		Thompkins and Binder, 2003 (2)	the 15 less and the 15 most skilled readers from 60 adults from ABE classes (age: 17-55)	30 control children chosen from a 99 pool (age: 5-8)	reading level	orthographic constraints
**Dyslexic Adults**	Deficit	Bogdanowicz et al., 2014	93 native Polish developmental dyslexic university students (age: 19-24)	87 native Polish university students (age: 20-24)		writing a short story: word structure errors

For explanation of (2), (3) see end of the table.

(Continued)

[14] Main effect of groups.
[15] Main effect of groups.
[16] Significant difference with CM2.
[17] Significant difference with CP, CM2.
[18] Significant difference with CP, CM2.
[19] Main effect of groups.

Supplementary Table S1 | Continued: Orthographic processing

Group	Performance	Study	Sample characteristics			Task
			Experimental	Control	Match	
**Dyslexic Adults**	Normal	Beidas et al., 2013	34 native Hebrew dyslexic university students (age: 21-28)	35 native Hebrew university students (age: 21-28)	chronological age, nonverbal IQ, verbal ability, handedness	parsing[20]
**Dyslexic Children**	Deficit	Suarez-Coalla et al., 2014	20 native Spanish children with developmental dyslexia (age: 7-10)	40 / 40 native Spanish control children (age: 7-11 / 6-7)	chronological age, gender, IQ, and socio-economic status / reading level, gender, IQ, and socio-economic status	read aloud unfamiliar words alone and within the context of a story, read aloud pseudo-words
**3. Lexical processing**						
**Illiterates**	Deficit	Kosmidis et al., 2006	19 illiterate adults (age: 63-92)	20 literate/low literate / 15 literate/high- educated adults with 1-9 / minimum 10 years of schooling (age: 56-85 / 55-74)	age, gender / gender	lexical decision
**Functional Illiterates**	Normal	Eme et al., 2010 (3)	52 native French ABE students (age: 17-55)	20 native French proficient readers (age: 18-52)	socio-economic status	producing an oral narrative based on 8 pictures: lexical diversity
		Eme et al., 2014 (3)	52 native French ABE students (age: 17-55)	52 native French children from 1st to 3rd grade	reading level	dictation: lexical errors
**Dyslexic Adults**	No data					

For explanation of (3) see end of the table.

(Continued)

[20] Significant difference in time but not in accuracy.

Supplementary Table S1 | Continued: Lexical processing

Group	Performance	Study	Sample characteristics			Task
			Experimental	Control	Match	
**Dyslexic Children**	Deficit	Martens and de Jong, 2006	22 dyslexic children (age: 9-10)	22 / 22 normal readers (age: 10 / 7-8)	age, gender, vocabulary, and nonverbal reasoning ability / reading level, gender	lexical decision
**4. Morphological awareness**						
**Illiterates**	No data					
**Functional Illiterates**	Deficit	Eme et al., 2010 (3)	52 native French ABE students (age: 17-55)	20 native French proficient readers (age: 18-52)	socio-economic status	producing an oral narrative based on 8 pictures: morphosyntactic errors
		Eme et al., 2014 (3)	52 native French ABE students (age: 17-55)	52 native French children from 1st to 3rd grade	reading level	morphosyntactic integration
	Normal	Eme et al., 2014 (3)	52 native French ABE students (age: 17-55)	52 native French children from 1st to 3rd grade	reading level	sentence recall
**Dyslexic Adults**	Deficit	Law et al., 2015	36 / 21 native English dyslexic / noncompensated university students (age: 17-26 / 17-28)	54 / 15 native English university students / compensated dyslexic university students (age: 19-25 / 20-23)	age, gender, education, non-verbal IQ / gender, non-verbal IQ	derivational suffix, nonword sentence completion
**Dyslexic Children**	Deficit	Joanisse et al., 2000	61 dyslexic children (age: 7-9)	52 / 37 non-dyslexic children (age: 7-9 / 6-8)	chronological age / reading level	infectional morphology

For explanation of (3) see end of the table.

(Continued)

Supplementary Table S1 | Continued: Morphological awareness

Group	Performance	Study	Sample characteristics			Task
			Experimental	Control	Match	
**Dyslexic Children**	Deficit	Casalis et al., 2004	33 native French dyslexic children (age: 8-12)	33 / 33 native French non-dyslexic children (mean age: 7.4 / 10.8)	chronological age / reading age	morphological analysis: segmentation, suffix deletion, derivation in sentence completion[21], production after definition[22], morphological fluency
		Chung et al., 2010	77 dyslexic students (age: 12-14)	27 normally achieving students (age: 12-14)	chronological age, IQ	morpheme discrimination, morpheme production
	Normal	Casalis et al., 2004	33 native French dyslexic children (age: 8-12)	33 / 33 native French non-dyslexic children (mean age: 7.4 / 10.8)	chronological age / reading age	morphological analysis: blending
		Chung et al., 2010	77 dyslexic students (age: 12-14)	27 normally achieving students (age: 9-13)	reading level, IQ	morpheme discrimination, morpheme production
**5. Spelling**						
**Illiterates**	No data					
**Functional Illiterates**	Deficit	Greenberg et al., 1997[23] (2)	72 native English adults from ABE classes (age: 21-45)	72 native English children from 3rd to 5th grade	gender, race, reading level	spelling inventory
		Thompkins and Binder, 2003 (2)	the 15 less and the 15 most skilled readers from 60 adults from ABE classes (age: 17-55)	30 control children chosen from a 99 pool (age: 5-8)	reading level	phonological spelling, orthographic spelling
		Eme et al., 2014 (3)	52 native French ABE students (age: 17-55)	52 native French children from 1st to 3rd grade	reading level	pseudo-word spelling, dictation

For explanation of (2), (3) see end of the table.
(Continued)

[21] Main effect of groups.
[22] Main effect of groups.
[23] Main effect of groups.

Supplementary Table S1 | Continued: Spelling

Group	Performance	Study	Sample characteristics			Task
			Experimental	Control	Match	
**Functional Illiterates**	Normal	Eme et al., 2014 (3)	52 native French ABE students (age: 17-55)	52 native French children from 1st to 3rd grade	reading level	dictation: word spelling, grammatical errors
**Dyslexic Adults**	Deficit	Hatcher et al., 2002	23 dyslexic university students (age: 19-52)	50 university students (age: 18-41)	verbal and non-verbal abilities	Wide Range Achievement Test of Spelling
		Beidas et al., 2013	34 native Hebrew dyslexic university students (age: 21-28)	35 native Hebrew university students (age: 21-28)	chronological age, nonverbal IQ, verbal ability, handedness	spelling
		Law et al., 2015	36 native English dyslexic university students (age: 17-26)	54 native English university students (age: 19-25)	age, gender, education, non-verbal IQ	spelling (Wechsler Individual Achievement Test-III)
		Wilson et al., 2015	18 / 22 / 26 native English dyslexic (a) / dyscalculic (b) / dyslexic & dyscalculic adults (c) (age: 30-32 / 27-31 / 28-32)	19 native English control adults (d) (age: 26-30)	age, gender, handedness, education, socio-economic status	regular and irregular word spelling [24]
	Normal	Law et al., 2015	21 native English noncompensated dyslexic university students (age: 17-28)	15 native English compensated dyslexic university students (age: 20-23)	gender, non-verbal IQ	spelling (Wechsler Individual Achievement Test-III)
**Dyslexic Children**	Deficit	White et al., 2006	23 dyslexic children (age: 8-12)	22 control children (age: 8-12)	age, gender, non-verbal IQ	spelling (Wide Range Achievement Test)
		Everatt et al., 2008	20 native English dyslexic children (age: 11-12)	40 native English control children (age: 11-12)	age, gender	word spelling (based on the Vernon Graded Word Spelling test)
		Chung et al., 2010	77 dyslexic students (age: 12-14)	27 / 27 normally achieving students (age: 12-14 / 9-13)	chronological age, IQ / reading level, IQ	Chinese word dictation

For explanation of (3) see end of the table.

(Continued)

[24] Significant difference between a and d.

Supplementary Table S1 | Continued: Vocabulary

Group	Performance	Study	Sample characteristics: Experimental	Sample characteristics: Control	Sample characteristics: Match	Task
**6. Vocabulary**						
**Illiterates**	No data					
**Functional Illiterates**	Deficit	Greenberg et al., 1997[25] (2)	72 native English adults from ABE classes (age: 21-45)	72 native English children from 3rd to 5th grade	gender, race, reading level	repetitive vocabulary (Peabody Picture Vocabulary Test-Revised)
		Eme, 2006 (3)[26]	50 native French functional illiterate adults (age: 17-55)	20 / 20 / 20 / 20 native French control children from preparatory class (CP) / "1st grade" (CE1) / "2nd grade" (CE2) / "4th grade" (CM2) (mean age: 6.7 / 7.6 / 8.8 / 10.7)		antonym[27] and vocabulary[28] (Wechsler Adult Intelligence Scale-III)
		Eme et al., 2014 (3)	52 native French ABE students (age: 17-55)	52 native French children from 1st to 3rd grade	reading level	antonym and vocabulary
**Dyslexic Adults**	Deficit	Law et al., 2015	21 native English noncompensated dyslexic university students (age: 17-28)	15 native English compensated dyslexic university students (age: 20-23)	gender, non-verbal IQ	word definition (Clinical Evaluation of Language Fundamentals Fourth Edition)
	Normal	Hatcher et al., 2002	23 dyslexic university students (age: 19-52)	50 university students (age: 18-41)	verbal and non-verbal abilities	vocabulary (Wechsler Adult Intelligence Scale-Revised)
		Wiseheart et al., 2009	23 native English adults with developmental dyslexia (age: 17-23)	33 native English university students (age: 18-23)	age	vocabulary (Wechsler Adult Intelligence Scale-III), Shipley Vocabulary Test

For explanation of (2), (3) see end of the table.

(Continued)

[25] Main effect of groups.
[26] Main effect of groups.
[27] Significant difference with CP.
[28] Significant difference with CP, CE1.

Supplementary Table S1 | Continued: Vocabulary

Group	Performance	Study	Sample characteristics			Task
			Experimental	Control	Match	
**Dyslexic Adults**	Normal	Cavalli et al., 2016	20 French native university students with dyslexia (age: 19-28)	20 French native university students (age: 19-28)	chronological age, gender, non-verbal IQ, level of education	vocabulary breadth (Echelle de Vocabulaire en Images Peabody), vocabulary depth (vocabulary subtest of Wechsler Adult Intelligence Scale-III)
**Dyslexic Children**	Deficit	Joanisse et al., 2000	61 dyslexic children (age: 7-9)	52 / 37 non-dyslexic children (age: 7-9 / 6-8)	chronological age / reading level	vocabulary (Wechsler Intelligence Scale for Children-III)
	Normal	Everatt et al., 2008	20 native English dyslexic children (age: 11-12)	40 native English control children (age: 11-12)	age, gender	British Picture Vocabulary Scale
**7. Reading/verbal fluency**						
**Illiterates**	Deficit	Rosselli et al., 1990[29]	100 native Spanish illiterates with no formal education and with illiterate parents	100 native Spanish professionals with professional parents	age, gender, handedness	semantic fluency (categories: animals, fruits)
		Reis and Castro-Caldas, 1997	20 illiterate adults	10 literate adults	gender, cultural and social background	semantic fluency (categories: animals, furnitures)
		Ostrosky-Solis et al., 1999[30]	199 native Spanish illiterates (age: 16-85)	199 / 201 / 201 native Spanish participants with 1-4 / 5-9 / 10-24 years of schooling (age: 16-85)		semantic fluency (category: animal)
		Kosmidis et al., 2004	19 illiterate adults (age: 63-92)	20 literate/low literate adults with 1-9 years of schooling (age: 56-85) / 21 literate/high- educated women with minimum 9 years of schooling (age: 55-74)	age, gender / gender	semantic fluency (categories: animals, objects, fruits)

(Continued)

[29] The authors divided the participants into groups according to 3 variables. Here we used the most relevant grouping variable: educational level.
[30] The authors ranked the participants into 16 groups according to age and years of education. Here we focus on the years of education.

Supplementary Table S1 | Continued: Reading/verbal fluency

Group	Performance	Study	Sample characteristics			Task
			Experimental	Control	Match	
**Illiterates**	Normal	Reis et al., 2003	23 completely illiterates (age: 57-76)	18 / 9 literates with 4 / more than 4 years of schooling (age: 51-76 / 56-69)	age, gender, general health, sociocultural background, level of everyday functionality	semantic fluency (category: supermarket)
**Functional Illiterates**	Deficit	Van Linden and Cremers, 2008 (1)	23 functional illiterate adults (age: 21-76)	23 literate adults (age: 19-64)	age, gender	reading and writing fluency
**Dyslexic Adults**	Normal	Hatcher et al., 2002	23 dyslexic university students (age: 19-52)	50 university students (age: 18-41)	verbal and non-verbal abilities	semantic fluency (categories: animals, food)
**Dyslexic Children**	Deficit	White et al., 2006	23 dyslexic children (age: 8-12)	22 control children (age: 8-12)	age, gender, non-verbal IQ	rhyme fluency (Phonological Assessment Battery)
		Varvara et al., 2014	60 children and adolescents with developmental dyslexia (age: 8-17)	65 children with typical reading abilities (age: 8-16)	chronological and mental age	category fluency (categories: e.g. animals, clothes, fruits, toys)
	Normal	White et al., 2006	23 dyslexic children (age: 8-12)	22 control children (age: 8-12)	age, gender, non-verbal IQ	alliteration, semantic fluency (Phonological Assessment Battery)
		Landerl et al., 2009	21 native German dyslexic children (a) (age: 7-10)	20 / 26 / 42 native German dyscalculic (b) / dyslexic & dyscalculic (c) / control children (d) (age: 7-10 / 7-11 / 7-10)	age	semantic fluency (category: animals)

For explanation of (1) see end of the table.

(Continued)

Supplementary Table S1 | Continued: Sentence comprehension

Group	Performance	Study	Sample characteristics			Task
			Experimental	Control	Match	
**8. Sentence comprehension**						
**Illiterates**	No data					
**Functional Illiterates**	Deficit	Eme, 2006 (3)[31]	50 native French functional illiterates (age: 17-55)	20 / 20 / 20 / 20 native French control children from preparatory class (CP) / "1st grade" (CE1) / "2nd grade" (CE2) / "4th grade" (CM2) (mean age: 6.7 / 7.6 / 8.8 / 10.7)		complete sentences[32] (Language Oral, Language Écrit, Mémoire, Attention)
**Dyslexic Adults**	Deficit	Wiseheart et al., 2009	23 native English adults with developmental dyslexia (age: 17-23)	33 native English university students (age: 18-23)	age	Kempler Sentence Comprehension Test
**Dyslexic Children**	Deficit	Rimrodt et al., 2009	14 children with learning disabilities/dyslexia (age: 9-14)	15 control children (age: 10-14)	age	sentence comprehension[33]
	Normal	Rimrodt et al., 2009	14 children with learning disabilities/dyslexia (age: 9-14)	15 control children (age: 10-14)	age	word recognition
**9. Reading comprehension**						
**Illiterates**	Deficit	Ostrosky-Solis et al., 1999[34]	199 native Spanish illiterate adults (age: 16-85)	199 / 201 / 201 native Spanish participants with 1-4 / 5-9 / 10-24 years of schooling (age: 16-85)		read a story aloud and answer some questions

For explanation of (3) see end of the table.

(Continued)

[31] Main effect of groups.
[32] Significant difference with CP, CM2.
[33] Significant difference in accuracy.
[34] The authors ranked the participants into 16 groups according to age and years of education. Here we focus on the years of education.

Supplementary Table S1 | Continued: Reading comprehension

Group	Performance	Study	Sample characteristics			Task
			Experimental	Control	Match	
**Functional Illiterates**	Deficit	Eme, 2006 (3)[35]	50 native French functional illiterate adults (age: 17-55)	20 / 20 / 20 / 20 native French control children from preparatory class (CP) / “1st grade” (CE1) / “2nd grade” (CE2) / “4th grade” (CM2) (mean age: 6.7 / 7.6 / 8.8 / 10.7)		written text comprehension[36]
	Normal	Eme et al., 2014 (3)	52 native French ABE students (age: 17-55)	52 native French children from 1st to 3rd grade	reading level	written comprehension
**Dyslexic Adults**	Deficit	Law et al., 2015	36 / 21 native English dyslexic / noncompensated university students (age: 17-26 / 17-28)	54 / 15 native English university students / compensated dyslexic university students (age: 19-25 / 20-23)	age, gender, education, non-verbal IQ / gender, non-verbal IQ	passage reading (Woodcock-Johnson III)
		Rello et al., 2013	23 native Spanish dyslexic adolescents and adults (age: 13-37)	23 native Spanish adolescents and adults (age: 13-35)		multiple choice text comprehension
		Beidas et al., 2013	34 native Hebrew dyslexic university students (age: 21-28)	35 native Hebrew university students (age: 21-28)	chronological age, nonverbal IQ, verbal ability, handedness	reading comprehension
**Dyslexic Children**	Deficit	Casalis et al., 2004	33 native French dyslexic children (age: 8-12)	33 / 33 native French non-dyslexic children (mean age: 7.4 / 10.8)	chronological age / reading age	syntactical comprehension (reading)
		Chung et al., 2010	77 dyslexic students (age: 12-14)	27 / 27 normally achieving students (age: 12-14 / 9-13)	chronological age, IQ / reading level, IQ	reading comprehension

For explanation of (3) see end of the table.

(Continued)

[35] Main effect of groups.
[36] Significant difference with CP, CM2.

Supplementary Table S1 | Continued: Oral comprehension

Group	Performance	Study	Sample characteristics			Task
			Experimental	Control	Match	
**10. Oral comprehension**						
**Illiterates**	Deficit	Rosselli et al., 1990[37]	100 native Spanish illiterates with no formal education and with illiterate parents	100 native Spanish professionals with professional parents	age, gender, handedness	language comprehension: verbal commands
		Ostrosky-Solis et al., 1999[38]	199 native Spanish illiterate adults (age: 16-85)	199 / 201 / 201 native Spanish participants with 1-4 / 5-9 / 10-24 years of schooling (age: 16-85)		language comprehension: verbal commands
	Normal	Reis et al., 2003	23 completely illiterate adults (age: 57-76)	18 / 9 literates with 4 / more than 4 years of schooling (age: 51-76 / 56-69)	age, gender, general health, sociocultural background, level of everyday functionality	oral language comprehension: verbal commands
**Functional Illiterates**	Deficit	Van Linden and Cremers, 2008 (1)	23 functional illiterate adults (age: 21-76)	23 literate adults (age: 19-64)	age, gender	listening
	Normal	Eme, 2006 (3)[39]	50 native French functional illiterate adults (age: 17-55)	20 / 20 / 20 / 20 native French control children from preparatory class (CP) / “1st grade” (CE1) / “2nd grade” (CE2) / “4th grade” (CM2) (mean age: 6.7 / 7.6 / 8.8 / 10.7)		oral comprehension
		Eme et al., 2014 (3)	52 native French ABE students (age: 17-55)	52 native French children from 1st to 3rd grade	reading level	oral comprehension

For explanation of (1), (3) see end of the table.

(Continued)

[37] The authors divided the participants into groups according to 3 variables. Here we used the most relevant grouping variable: educational level.
[38] The authors ranked the participants into 16 groups according to age and years of education. Here we focus on the years of education.
[39] Main effect of groups.

Supplementary Table S1 | Continued: Oral comprehension

Group	Performance	Study	Sample characteristics			Task
			Experimental	Control	Match	
**Dyslexic Adults**	No data					
**Dyslexic Children**	Deficit	Willcutt et al, 2013	241 /183 / 188 children with reading disability (a) / math disability (b) / reading & math disability (c) (age: 8-13 / 9-13/ 8-13)	411 control children (d) (age: 8-13)	age, gender, ethnicity	Verbal comprehension (Wechsler Intelligence Scale for Children, Revised)[40]
	Normal	Everatt et al., 2008	20 native English dyslexic children (age: 11-12)	40 native English control children (age: 11-12)	age, gender	listening comprehension
		Casalis et al., 2004	33 native French dyslexic children (age: 8-12)	33 / 33 native French non-dyslexic children (mean age: 7.4 / 10.8)	chronological age / reading age	syntactical comprehension (listening)
**11. Naming ability**						
**Illiterates**	Deficit	Rosselli et al., 1990[41]	100 native Spanish illiterates with no formal education and with illiterate parents	100 native Spanish professionals with professional parents	age, gender, handedness	object, figure, body-part naming
		Ostrosky-Solis et al., 1999[42]	199 native Spanish illiterate adults (age: 16-85)	199 / 201 / 201 native Spanish participants with 1-4 / 5-9 / 10-24 years of schooling (age: 16-85)		name line drawing figures
		Reis et al., 2006	19 illiterate adults (age: 61-75)	19 literate adults (age: 56-83)	age, gender, socio-cultural background	immediate object naming: colored and black and white photos and drawings

(Continued)

[40] Significant difference between a, c and d, and b, c and d.
[41] The authors divided the participants into groups according to 3 variables. Here we used the most relevant grouping variable: educational level.
[42] The authors ranked the participants into 16 groups according to age and years of education. Here we focus on the years of education.

Supplementary Table S1 | Continued: Naming ability

Group	Performance	Study	Sample characteristics			Task
			Experimental	Control	Match	
**Illiterates**	Normal	Reis et al., 2003	23 completely illiterate adults (age: 57-76)	18 / 9 literates with 4 / more than 4 years of schooling (age: 51-76 / 56-69)	age, gender, general health, sociocultural background, level of everyday functionality	visual naming: real objects
**Functional Illiterates**	Deficit	Grosche, 2012 (3)	54 ABE students (age: 33-53)	66 native German control adults (age: 32-53)	chronological age	color, object and letter naming
	Normal	Grosche, 2012 (3)	54 ABE students (age: 33-53)	54 children from 1st to 4th grade (age: 7-9)	reading level	color, object and letter naming
**Dyslexic Adults**	Deficit	Hatcher et al., 2002	23 dyslexic university students (age: 19-52)	50 university students (age: 18-41)	verbal and non-verbal abilities	digit and object naming (Phonological Assessment Battery)
		Ramus et al., 2003	16 dyslexic university students (age: 19-22)	16 university students (age: 19-24)	age, gender, handedness, full-scale IQ	automatic picture naming
		De Smedt and Boets, 2011	25 dyslexic university students (age: 18-28)	25 normal reading controls (age: 18-29)	age, nonverbal IQ	color, object and letter naming
		Beidas et al., 2013	34 native Hebrew dyslexic university students (age: 21-28)	35 native Hebrew university students (age: 21-28)	chronological age, nonverbal IQ, verbal ability, handedness	naming speed test: objects
		Bogdanowicz et al., 2014	93 native Polish developmental dyslexic university students (age: 19-24)	87 native Polish university students (age: 20-24)		Rapid Automatized Naming test: objects
		Law et al., 2015	36 native English dyslexic university students (age: 17-26)	54 native English university students (age: 19-25)	age, gender, education, non-verbal IQ	color and object naming (Phonological Assessment Battery)

For explanation of (3) see end of the table.
(Continued)

Supplementary Table S1 | Continued: Naming ability

Group	Performance	Study	Sample characteristics			Task
			Experimental	Control	Match	
**Dyslexic Adults**	Deficit	Wilson et al., 2015	18 / 22 / 26 native English dyslexic (a) / dyscalculic (b) / dyslexic & dyscalculic adults (c) (age: 30-32 / 27-31 / 28-32)	19 native English control adults (d) (age: 26-30)	age, gender, handedness, education, socio-economic status	Digit and letter naming (Comprehensive Test of Phonological Processing)[43]
	Normal	Bogdanowicz et al., 2014	93 native Polish developmental dyslexic university students (age: 19-24)	87 native Polish university students (age: 20-24)		Rapid Automatized Naming test: symbols
**Dyslexic Children**	Deficit	White et al., 2006	23 dyslexic children (age: 8-12)	22 control children (age: 8-12)	age, gender, non-verbal IQ	naming speed: pictures, digits (Phonological Assessment Battery)
		Everatt et al., 2008	20 native English dyslexic children (age: 11-12)	40 native English control children (age: 11-12)	age, gender	color naming, Stroop incongruous color words, incongruous colored objects
		Willburger et al., 2008	18 / 19 / 20 native German dyslexic (a) / dyscalculic (b) / dyslexic & dyscalculic (c) children (age: 8-10)	42 native German control children (d) (age: 8-9)	age, arithmetic score / age, reading score / age	digit[44], letter[45] and object naming[46]
		Boets and De Smedt, 2010	13 native Dutch dyslexic children (age: 8)	16 native Dutch control children (age: 8)	gender, parental educational level, intellectual ability	digit naming
		Chung et al., 2010	77 dyslexic students (age: 12-14)	27 normally achieving students (age: 12-14)	chronological age, IQ	digit and letter naming

(Continued)

[43] Significant difference between a, b, c and d.
[44] Significant difference between a and b, d; c and a, b, d.
[45] Significant difference between a and d; c and b, d.
[46] Significant difference between c and d.

Supplementary Table S1 | Continued: Naming ability

Group	Performance	Study	Sample characteristics			Task
			Experimental	Control	Match	
**Dyslexic Children**	Deficit	Willcutt et al, 2013	241 /183 / 188 children with reading disability (a) / math disability (b) / reading & math disability (c) (age: 8-13 / 9-13/ 8-13)	411 control children (d) (age: 8-13)	age, gender, ethnicity	Rapid Automatized Naming test: objects, numbers, letters, colors[47]
	Normal	Landerl et al., 2009	21 native German dyslexic children (a) (age: 7-10)	20 / 26 / 42 native German dyscalculic (b) / dyslexic & dyscalculic (c) / control children (d) (age: 7-10 / 7-11 / 7-10)	age	digit naming
		Chung et al., 2010	77 dyslexic students (age: 12-14)	27 normally achieving students (age: 9-13)	reading level, IQ	digit and letter naming
**12. Reading speed**						
**Illiterates**	No data					
**Functional Illiterates**	No data					
**Dyslexic Adults**	Deficit	Ramus et al., 2003	16 dyslexic university students (age: 19-22)	16 university students (age: 19-24)	age, gender, handedness, full-scale IQ	National Adult Reading Test
		Rello et al., 2013	23 native Spanish dyslexic adolescents and adults (age: 13-37)	23 native Spanish adolescents and adults (age: 13-35)		reading speed of 4 short texts

(Continued)

[47] Significant difference between a, b, c and d.

Supplementary Table S1 | Continued: Reading speed

Group	Performance	Study	Sample characteristics			Task
			Experimental	Control	Match	
**Dyslexic Children**	Deficit	Suarez-Coalla et al., 2014	20 native Spanish children with developmental dyslexia (age: 7-10)	40 / 40 native Spanish control children (age: 7-11 / 6-7)	chronological age, gender, IQ, and socio-economic status / reading level, gender, IQ, and socio-economic status	read aloud unfamiliar words alone and within the context of a story, read aloud pseudo-words

(1) Sample is termed functional illiterate, but no reason/explanation/diagnostic justification is given
(2) Experiments on ABE students who are (sometimes) named as functional illiterates
(3) Experiments on ABE students who are identified as functional illiterates

Supplementary Table S2 | Summary of cognitive deficits of illiterates, functional illiterates, dyslexic adults and children

Group	Performance	Study	Sample characteristics			Task
			Experimental	Control	Match	
**1. Working memory**						
**Illiterates**	Deficit	Ardila et al., 1989[48]	100 native Spanish illiterates with no formal education and with illiterate parents (age: 16-65)	100 native Spanish professionals with professional parents (age: 16-65)	age, gender, handedness	digit retention: forward and backward
		Reis et al., 2003	23 completely illiterates (age: 57-76)	18 / 9 literates with 4 / more than 4 years of schooling (age: 51-76 / 56-69)	age, gender, general health, sociocultural background, level of everyday functionality	digit span: forward (Wechsler Memory Scale)
		Kosmidis et al., 2011	20 illiterate women (age: 62-76)	12 functional illiterate adults / 6 self-educated women / 27 educated literate adults (age: 61-73 / 60-74 / 58-72)	age, general cognitive status, depression	digit span: forward, backward (Wechsler Adult Intelligence Scale-III), sentence span, spatial span: backward (Wechsler Adult Intelligence Scale-III)
		Silva et al., 2012	19 illiterate adults (age: 65-71)	19 literate adults (age: 59-73)	age, gender	digit span: forward, backward (Wechsler Memory Scale-III)
	Normal	Kosmidis et al., 2011	20 illiterate women (age: 62-76)	12 functional illiterate adults / 6 self-educated women / 27 educated literate adults (age: 61-73 / 60-74 / 58-72)	age, general cognitive status, depression	spatial span: forward (Wechsler Adult Intelligence Scale-III)
**Functional Illiterates**	Deficit	Thompkins and Binder, 2003 (2)	the 15 less and the 15 most skilled readers from 60 adults from ABE classes (age: 17-55)	30 control children chosen from a 99 pool (age: 5-8)	reading level	digit span: backward

For explanation of (2) see end of the table.

(Continued)

[48] The authors divided the participants into groups according to 3 variables. Here we used the most relevant grouping variable: educational level.

Supplementary Table S2 | Continued: Working memory

Group	Performance	Study	Sample characteristics			Task
			Experimental	Control	Match	
**Functional Illiterates**	Deficit	Eme, 2006 (3)[49]	50 native French functional illiterate adults (age: 17-55)	20 / 20 / 20 / 20 native French control children from preparatory class (CP) / "1st grade" (CE1) / "2nd grade" (CE2) / "4th grade" (CM2) (mean age: 6.7 / 7.6 / 8.8 / 10.7)		digit span: forwards[50], backward[51] **(**Wechsler Adult Intelligence Scale-III**)**
		Grosche, 2012 (3)	54 ABE students (age: 33-53)	66 native German control adults / 54 children from 1st to 4th grade (age: 32-53 / 7-9)	chronological age / reading level	word span (1 syllable words), word span (3 syllable words)[52], pseudo-word repetition (Arbeitsgedächtnistestbatterie für Kinder von 5 bis 12 Jahren)
	Normal	Grosche, 2012 (3)	54 ABE students (age: 33-53)	54 children from 1st to 4th grade (age: 7-9)	reading level	word span (3 syllable words) (Arbeitsgedächtnistestbatterie für Kinder von 5 bis 12 Jahren)
		Thompkins and Binder, 2003 (2)	the 15 less and the 15 most skilled readers from 60 adults from ABE classes (age: 17-55)	30 control children chosen from a 99 pool (age: 5-8)	reading level	digit span: forward
**Dyslexic Adults**	Deficit	Hatcher et al., 2002	23 dyslexic university students (age: 19-52)	50 university students (age: 18-41)	verbal and non-verbal abilities	digit span: forward, backward (Wechsler Adult Intelligence Scale-Revised)

For explanation of (2), (3) see end of the table.
(Continued)

[49] Main effect groups.
[50] Significant difference with CE1, CM2.
[51] Significant difference with CM2.
[52] Significant difference between the adult groups.

Supplementary Table S2 | Continued: Working memory

Group	Performance	Study	Sample characteristics			Task
			Experimental	Control	Match	
**Dyslexic Adults**	Deficit	Brosnan et al., 2002	9 dyslexic university students (age: 22-45)	9 university students (age: 22-37)	gender, age, academic year and major, current academic grades, socio- economic status	digit span: forward, backward (Wechsler Adult Intelligence Scale-Revised)
		Brosnan et al., 2002	15 dyslexic university students (age: 19-26)	15 university students (age: 23-28)	gender, age, academic year and major, current academic grades, socio-economic status	digit span: forward, backward (Wechsler Adult Intelligence Scale-Revised)
		Wiseheart et al., 2009	23 native English adults with developmental dyslexia (age: 17-23)	33 native English university students (age: 18-23)	age	digit span: forward, backward (Wechsler Memory Scale), digit ordering
		Abd Ghani and Gathercole, 2013	26 dyslexic university students	32 university students		listening recall, backward digit span, odd one out, spatial recall (Automated Working Memory Assessment)
		Beidas et al., 2013	34 native Hebrew dyslexic university students (age: 21-28)	35 native Hebrew university students (age: 21-28)	chronological age, nonverbal IQ, verbal ability, handedness	Neuropsychological Examination CogniFit Personal Coach: auditory verbal, visual
		Bogdanowicz et al., 2014	93 native Polish developmental dyslexic university students (age: 19-24)	87 native Polish university students (age: 20-24)		digit span (Wechsler Memory Scale III)
		Law et al., 2015	36 native English dyslexic university students (age: 17-26)	54 native English university students (age: 19-25)	age, gender, education, non-verbal IQ	digit span: forward (Clinical Evaluation of Language Fundamentals Fourth Edition), nonword recall (Working Memory Test Battery)

(Continued)

Supplementary Table S2 | Continued: Working memory

Group	Performance	Study	Sample characteristics			Task
			Experimental	Control	Match	
**Dyslexic Adults**	Deficit	Smith-Spark et al., 2016	31 dyslexic university students (age: 19-30)	30 control university students (age: 18-29)	age, IQ	operation span, symmetry span
	Normal	Brosnan et al., 2002	15 dyslexic university students (age: 19-26)	15 university students (age: 23-28)	gender, age, academic year and major, current academic grades, socio-economic status	spatial span (Cambridge Neuropsychological Test Automated Battery)
		Beidas et al., 2013	34 native Hebrew dyslexic university students (age: 21-28)	35 native Hebrew university students (age: 21-28)	chronological age, nonverbal IQ, verbal ability, handedness	Neuropsychological Examination CogniFit Personal Coach: auditory non-verbal
**Dyslexic Children**	Deficit	Brosnan et al., 2002	16 dyslexic children (age: 9-10)	16 non-dyslexic children (age: 9-10)	age, gender	digit span: silent, noisy (Wechsler Adult Intelligence Scale-III)
		Everatt et al., 2008	20 native English dyslexic children (age: 11-12)	40 native English control children (age: 11-12)	age, gender	digit span: forward, reverse (Wechsler Intelligence Scale for Children)
		Landerl et al., 2009	21 native German dyslexic children (a) (age: 7-10)	20 / 26 / 42 native German dyscalculic (b) / dyslexic & dyscalculic (c) / control children (d) (age: 7-10 / 7-11 / 7-10)	age	digit span: backward (Wechsler Intelligence Scale for Children-III)[53], nonword span[54], Corsi blocks [55]
		Beneventi et al., 2010	12 native Norwegian dyslexic children (age: 13-14)	14 native Norwegian control children (age: 12-13)	age, gender, non-verbal IQ	n-back task with letters: 0-back, 1-back, 2-back[56]

(Continued)

[53] Significant difference between a and c.
[54] Significant difference between a and d.
[55] Significant difference between a and c.
[56] Significant main effect of group on accuracy (1-back, 2-back) and on reaction time (0-back, 1-back, 2-back).

Supplementary Table S2 | Continued: Working memory

Group	Performance	Study	Sample characteristics			Task
			Experimental	Control	Match	
**Dyslexic Children**	Deficit	Chung et al., 2010	77 dyslexic students (age: 12-14)	27 / 27 normally achieving students (age: 12-14 / 9-13)	chronological age, IQ / reading level, IQ	digit span: backward
		Willcutt et al, 2013	241 /183 / 188 children with reading disability (a) / math disability (b) / reading & math disability (c) (age: 8-13 / 9-13/ 8-13)	411 control children (d) (age: 8-13)	age, gender, ethnicity	sentence span, counting span, digit span: backwards (Wechsler Intelligence Scale for Children, Revised)[57]
		Varvara et al., 2014	60 children and adolescents with developmental dyslexia (age: 8-17)	65 children with typical reading abilities (age: 8-16)	chronological and mental age	verbal span, non-word repetition, visual span
	Normal	Landerl et al., 2009	21 native German dyslexic children (a) (age: 7-10)	20 / 26 / 42 native German dyscalculic (b) / dyslexic & dyscalculic (c) / control children (d) (age: 7-10 / 7-11 / 7-10)	age	digit span: forward (Wechsler Intelligence Scale for Children-III)
		Varvara et al., 2014	60 children and adolescents with developmental dyslexia (age: 8-17)	65 children with typical reading abilities (age: 8-16)	chronological and mental age	visual-spatial span
**2. Attention**						
**Illiterates**	Deficit	Ostrosky-Solis et al., 1999[58]	199 native Spanish illiterate adults (age: 16-85)	199 / 201 / 201 native Spanish participants with 1-4 / 5-9 / 10-24 years of schooling (age: 16-85)		digits backwards, visual detection, serial 3 substraction
		Landgraf et al., 2011	47 illiterate non-native participants	41 literate native German (except 3) participants	age, gender, handedness	d2

(Continued)

[57] Significant difference between a and c, d; b and c, d.

[58] The authors ranked the participants into 16 groups according to age and years of education. Here we focus on the years of education.

Supplementary Table S2 | Continued: Attention

Group	Performance	Study	Sample characteristics			Task
			Experimental	Control	Match	
**Functional Illiterates**	Deficit	Van Linden and Cremers, 2008 (1)	23 functional illiterate adults (age: 21-76)	23 literate adults (age: 19-64)	age, gender	steer a moving figure
	Normal	Eme, 2006 (3)[59]	50 native French functional illiterate adults (age: 17-55)	20 / 20 / 20 / 20 native French control children from preparatory class (CP) / "1st grade" (CE1) / "2nd grade" (CE2) / "4th grade" (CM2) (mean age: 6.7 / 7.6 / 8.8 / 10.7)		selective attention[60]
**Dyslexic Adults**	Deficit	Beidas et al., 2013	34 native Hebrew dyslexic university students (age: 21-28)	35 native Hebrew university students (age: 21-28)	chronological age, nonverbal IQ, verbal ability, handedness	sustained attention, divided attention, avoiding distracters (Neuropsychological Examination CogniFit Personal Coach)
		Bogdanowicz et al., 2014	93 native Polish developmental dyslexic university students (age: 19-24)	87 native Polish university students (age: 20-24)		difficult figure-copying
**Dyslexic Children**	Deficit	Zoubrinetzky et al., 2014	14 / 14 native French dyslexic children with visual attention span difficulties (a) / phonological difficulties (b) (age: 8-12 / 8-11)	14 / 14 native French control children (c) / (d) (age: 8-11 / 6-7)	(c) chronological age / (d) reading age	visual attention span: global and partial letter report[61]
		Varvara et al., 2014	60 children and adolescents with developmental dyslexia (age: 8-17)	65 children with typical reading abilities (age: 8-16)	chronological and mental age	map mission (Test of Everyday Attention for Children), code transmission (Test of Everyday Attention for Children)

For explanation of (1), (3) see end of the table.

(Continued)

[59] Main effect of groups.
[60] Significant difference with CP, CE1, CE2.
[61] Significant difference between a and b, c, d; b and a, d; c and a, d; d and a, b, c.

Supplementary Table S2 | Continued: Attention

Group	Performance	Study	Sample characteristics			Task
			Experimental	Control	Match	
**Dyslexic Children**	Normal	Willburger et al., 2008	18 / 19 / 20 native German dyslexic (a) / dyscalculic (b) / dyslexic & dyscalculic (c) children (age: 8-10)	42 native German control children (d) (age: 8-9)	age, arithmetic score / age, reading score / age	alertness, flexibility, sustained attention (Test of Attention Performance for Children)
**3. Perception**						
**Illiterates**	Deficit	Ardila et al., 1989[62]	100 native Spanish illiterates with no formal education and with illiterate parents (age: 16-65)	100 native Spanish professionals with professional parents (age: 16-65)	age, gender, handedness	immediate reproduction of the Rey-Osterrieth complex figure and of a cube and a house
		Ostrosky-Solis et al., 1999[63]	199 native Spanish illiterate adults (age: 16-85)	199 / 201 / 201 native Spanish participants with 1-4 / 5-9 / 10-24 years of schooling (age: 16-85)		copy of a semicomplex figure
		Dansilio and Charamelo, 2005	15 illiterate adults (age: 31-79)	15 literate adults	age, gender, handedness	figure copying: triangle, diamond, cube, house
**Functional Illiterates**	Deficit	Van Linden and Cremers, 2008 (1)	23 functional illiterate adults (age: 21-76)	23 literate adults (age: 19-64)	age, gender	copy the Rey complex figure
		Rüsseler et al., 2011 (3)	30 / 30 native German functional illiterate adults (age: 19-58 / 22-67)	30 / 30 native German normal readers / 30 native German children with reading and writing disabilities (age: 18-55 / 19-69 / 7-14)	age, IQ / age / -	visual and auditory order threshold, spatial hearing, auditory frequency and time pattern recognition, pitch discrimination, auditory motor coordination, choice reaction time (Brain-Boy)

For explanation of (1), (3) see end of the table.

(Continued)

[62] The authors divided the participants into groups according to 3 variables. Here we used the most relevant grouping variable: educational level.
[63] The authors ranked the participants into 16 groups according to age and years of education. Here we focus on the years of education.

Supplementary Table S2 | Continued: Perception

Group	Performance	Study	Sample characteristics			Task
			Experimental	Control	Match	
**Dyslexic Adults**	Deficit	Ramus et al., 2003	16 dyslexic university students (age: 19-22)	16 university students (age: 19-24)	age, gender, handedness, full-scale IQ	temporal order
		Leong et al., 2011	20 native English dyslexic adults (age: 17-41)	20 native English control adults (age: 18-38)	chronological age	amplitude envelope onset (rise time), frequency, intensity, syllable stress, word stress
		Bogdanowicz et al., 2014	93 native Polish developmental dyslexic university students (age: 19-24)	87 native Polish university students (age: 20-24)		difficult figure-copying
		Beidas et al., 2013	34 native Hebrew dyslexic university students (age: 21-28)	35 native Hebrew university students (age: 21-28)	chronological age, nonverbal IQ, verbal ability, handedness	visual perception (Neuropsychological Examination CogniFit Personal Coach)
	Normal	Ramus et al., 2003	16 dyslexic university students (age: 19-22)	16 university students (age: 19-24)	age, gender, handedness, full-scale IQ	auditory: backward and simultaneous masking, formant discrimination in syllables and non-speech analogues, phonemic categorization, frequency modulation detection;<br>visual: visual acuity, contrast sensitivity, speed discrimination, coherent motion detection
**Dyslexic Children**	Deficit	Ziegler et al., 2010	28 dyslexic children (age: 8-12)	29 normally developing children (age: 8-11)	chronological age, nonverbal IQ	perception of letter and digit strings

(Continued)

Supplementary Table S2 | Continued: Perception

Group	Performance	Study	Sample characteristics			Task
			Experimental	Control	Match	
**Dyslexic Children**	Deficit	Willcutt et al, 2013	241 /183 / 188 children with reading disability (a) / math disability (b) / reading & math disability (c) (age: 8-13 / 9-13/ 8-13)	411 control children (d) (age: 8-13)	age, gender, ethnicity	symbol search, coding (Wechsler Intelligence Scale for Children-III), Colorado Perceptual Speed Test, Educationnal Testing Service Identical Pictures Subtest[64]
	Normal	Joanisse et al., 2000	61 dyslexic children (age: 7-9)	52 / 37 non-dyslexic children (age: 7-9 / 6-8)	chronological age / reading level	single stimulus categorization
		Ziegler et al., 2010	28 dyslexic children (age: 8-12)	29 normally developing children (age: 8-11)	chronological age, nonverbal IQ	perception of symbol strings
**4. Executive functions**						
**Illiterates**	No data					
**Functional Illiterates**	No data					
**Dyslexic Adults**	Deficit	Brosnan et al., 2002	9 dyslexic university students (age: 22-45)	9 university students (age: 22-37)	gender, age, academic year and major, current academic grades, socio-economic status	group-embedded figures
		Beidas et al., 2013	34 native Hebrew dyslexic university students (age: 21-28)	35 native Hebrew university students (age: 21-28)	chronological age, nonverbal IQ, verbal ability, handedness	planning measure, shifting attention measure, inhibition measure (Neuropsychological Examination CogniFit Personal Coach)

(Continued)

[64] Significant difference between a and c, d; b and c, d.

Supplementary Table S2 | Continued: Executive functions

Group	Performance	Study	Sample characteristics			Task
			Experimental	Control	Match	
**Dyslexic Adults**	Deficit	Smith-Spark et al., 2016	31 dyslexic university students (age: 19-30)	30 control university students (age: 18-29)	age, IQ	plus-minus, inhibition[65]
	Normal	Brosnan et al., 2002	9 dyslexic university students (age: 22-45)	9 university students (age: 22-37)	gender, age, academic year and major, current academic grades, socio-economic status	stockings of Cambridge (Cambridge Neuropsychological Test Automated Battery), picture arrangement (Wechsler Adult Intelligence Scale-III)
		Smith-Spark et al., 2016	31 dyslexic university students (age: 19-30)	30 control university students (age: 18-29)	age, IQ	inhibition[66]
**Dyslexic Children**	Deficit	Brosnan et al., 2002	30 dyslexic children (age: 13-14)	30 non-dyslexic children (age: 13-14)	age, gender, demographic variables, socio-economic status, academic performance	group-embedded figures
		Willcutt et al., 2013	241 /183 / 188 children with reading disability (a) / math disability (b) / reading & math disability (c) (age: 8-13 / 9-13/ 8-13)	411 control children (d) (age: 8-13)	age, gender, ethnicity	inhibition: stop-signal task, Gordon Diagnostic System[67], set shifting: Wisconsin Card Sorting Test[68], inference control: Stroop (color and word)[69]

(Continued)

65 Significant difference for the accuracy of non-habituated stimuli.
66 Non-significant difference for accuracy and reaction time of habituated stimuli, and for reaction time of non-habituated stimuli.
67 Significant difference between d and a, b, c.
68 Significant difference between a and b, c; b and a, c; c and a, d; d and b, c.
69 Significant difference between a and b, d; b and a, c, d; c and b, d; d and a, b, c.

Supplementary Table S2 | Continued: Executive functions

Group	Performance	Study	Sample characteristics			Task
			Experimental	Control	Match	
**Dyslexic Children**	Normal	Varvara et al., 2014	60 children and adolescents with developmental dyslexia (age: 8-17)	65 children with typical reading abilities (age: 8-16)	chronological and mental age	Wisconsin Card Sorting Test
**5. Motor functions**						
**Illiterates**	Deficit	Rosselli et al., 1990[70]	100 native Spanish illiterates with no formal education and with illiterate parents	100 native Spanish professionals with professional parents	age, gender, handedness	buccofacial praxis, ideomotor praxis, finger alternating movements, meaningless movements, coordinated movements with both hands, motor impersistence
		Ostrosky-Solis et al., 1999[71]	199 native Spanish illiterate adults (age: 16-85)	199 / 201 / 201 native Spanish participants with 1-4 / 5-9 / 10-24 years of schooling (age: 16-85)		changing the position of the hand, alternating hand movements, opposite reactions
	Normal	Reis et al., 2003	23 completely illiterate adults (age: 57-76)	18 / 9 literates with 4 / more than 4 years of schooling (age: 51-76 / 56-69)	age, gender, general health, sociocultural background, level of everyday functionality	buccofacial, symbolic and limb ideomotor gestures
**Functional Illiterates**	No data					
**Dyslexic Adults**	Deficit	Brookes et al, 2010	20 dyslexic adults (age: 18-26)	30 university students (age: 20-23)	IQ, age	blindfolded heel-to-toe, “hold your arms”

(Continued)

[70] The authors divided the participants into groups according to 3 variables. Here we used the most relevant grouping variable: educational level.
[71] The authors ranked the participants into 16 groups according to age and years of education. Here we focus on the years of education.

Supplementary Table S2 | Continued: Motor functions

Group	Performance	Study	Sample characteristics			Task
			Experimental	Control	Match	
**Dyslexic Adults**	Normal	Ramus et al., 2003	16 dyslexic university students (age: 19-22)	16 university students (age: 19-24)	age, gender, handedness, full-scale IQ	balance/dual, bead threading, finger-to-thumb, repetitive finger-tapping, bimanual finger-tapping
		Brookes et al, 2010	20 dyslexic adults (age: 18-26)	30 university students (age: 20-23)	age, IQ	eyes-open heel-to-toe, "hold your arms"
**Dyslexic Children**	Deficit	White et al., 2006	23 dyslexic children (age: 8-12)	22 control children (age: 8-12)	age, gender, non-verbal IQ	stork balance, heel-to-toe
		Brookes et al, 2010	16 dyslexic children (age: 11-13)	25 control children (age: 11-13)	age, IQ	eyes-open and blindfolded heel-to-toe, "hold your arms"
	Normal	Everatt et al., 2008	20 native English dyslexic children (age: 11-12)	40 native English control children (age: 11-12)	age, gender	bead threading (based on the Dyslexia Screening Test)
		White et al., 2006	23 dyslexic children (age: 8-12)	22 control children (age: 8-12)	age, gender, non-verbal IQ	bead threading, finger and thumb

(1) Sample is termed functional illiterate, but no reason/explanation/diagnostic justification is given
(2) Experiments on ABE students who are (sometimes) named as functional illiterates
(3) Experiments on ABE students who are identified as functional illiterates

Supplementary Table S3 | Summary of mathematical related deficits of illiterates, functional illiterates, dyslexic adults and children

Group	Performance	Study	Sample characteristics			Task
			Experimental	Control	Match	
**Arithmetic abilities**						
**Illiterates**	Deficit	Rosselli et al., 1990[72]	100 native Spanish illiterates with no formal education and with illiterate parents	100 native Spanish professionals with professional parents	age, gender, handedness	basic mental calculations
		Ostrosky-Solis et al., 1999[73]	199 native Spanish illiterate adults (age: 16-85)	199 / 201 / 201 native Spanish participants with 1-4 / 5-9 / 10-24 years of schooling (age: 16-85)		simple arithmetic problems
		Reis et al., 2003	23 completely illiterate adults (age: 57-76) (sample 1)	18 / 9 literates with 4 / more than 4 years of schooling (age: 51-76 / 56-69)	age, gender, general health, sociocultural background, level of everyday functionality	mental calculation: addition, subtraction and multiplication
**Functional Illiterates**	No data					
**Dyslexic Adults**	Deficit	Hatcher et al., 2002	23 dyslexic university students (age: 19-52)	50 university students (age: 18-41)	verbal and non-verbal abilities	orally presented additions, subtractions (Graded Difficulty Arithmetic Test)
		De Smedt and Boets, 2011	25 dyslexic university students (age: 18-28)	25 normal reading controls (age: 18-29)	age, nonverbal IQ	subtractions, multiplications
	Normal	Simmons and Singleton, 2006[74]	19 dyslexic university students (age: 19-22)	19 university students (age: 19-21)	age, IQ	maths suite numbers and number facts: addition, subtraction, multiplication (LADS Memory test)

(Continued)

[72] The authors divided the participants into groups according to 3 variables. Here we used the most relevant grouping variable: educational level.
[73] The authors ranked the participants into 16 groups according to age and years of education. Here we focus on the years of education.
[74] Non-significant difference in accuracy but significant difference in speed.

Supplementary Table S3 | Continued: Arithmetic abilities

Group	Performance	Study	Sample characteristics			Task
			Experimental	Control	Match	
**Dyslexic Adults**	Normal	De Smedt and Boets, 2011	25 dyslexic university students (age: 18-28)	25 normal reading controls (age: 18-29)	age, nonverbal IQ	non-symbolic magnitude comparison
		Wilson et al., 2015	18 / 22 / 26 native English dyslexic (a) / dyscalculic (b) / dyslexic & dyscalculic adults (c) (age: 30-32 / 27-31 / 28-32)	19 native English control adults (d) (age: 26-30)	age, gender, handedness, education, socio-economic status	enumeration, number comparison, numerosity comparison, number line estimation, multiplication, subtraction[75]
**Dyslexic Children**	Deficit	Boets and De Smedt, 2010	13 native Dutch dyslexic children (age: 8)	16 native Dutch control children (age: 8)	gender, parental educational level, intellectual ability	single-digit multiplication and subtraction
	Normal	Landerl et al., 2009	21 native German dyslexic children (a) (age: 7-10)	20 / 26 / 42 native German dyscalculic (b) / dyslexic & dyscalculic (c) / control children (d) (age: 7-10 / 7-11 / 7-10)	age	symbolic magnitude comparison, non-symbolic magnitude comparison, number line estimation

[75] Significant difference between b and d in all tasks except in numerosity comparison.
